# TFEB controls integrin-mediated endothelial cell adhesion by the regulation of cholesterol metabolism

**DOI:** 10.1007/s10456-022-09840-x

**Published:** 2022-05-11

**Authors:** Camilla Ariano, Chiara Riganti, Davide Corà, Donatella Valdembri, Giulia Mana, Elena Astanina, Guido Serini, Federico Bussolino, Gabriella Doronzo

**Affiliations:** 1 Department of Oncology, University of Torino, Candiolo, Italy; 2 Candiolo Cancer Institute- FPO-IRCCS, Candiolo, Italy; 3 Department of Oncology, University of Torino, Torino, Italy; 4 Department of Translational Medicine, Piemonte Orientale University, Novara, Italy; 5 Center for Translational Research on Autoimmune and Allergic Diseases—CAAD, Novara, Italy

**Keywords:** TFEB, Endothelial cells, Cell adhesion, Integrin, Cholesterol

## Abstract

The dynamic integrin-mediated adhesion of endothelial cells (ECs) to the surrounding ECM is fundamental for angiogenesis both in physiological and pathological conditions, such as embryonic development and cancer progression. The dynamics of EC-to-ECM adhesions relies on the regulation of the conformational activation and trafficking of integrins. Here, we reveal that oncogenic transcription factor EB (TFEB), a known regulator of lysosomal biogenesis and metabolism, also controls a transcriptional program that influences the turnover of ECM adhesions in ECs by regulating cholesterol metabolism. We show that TFEB favors ECM adhesion turnover by promoting the transcription of genes that drive the synthesis of cholesterol, which promotes the aggregation of caveolin-1, and the caveolin-dependent endocytosis of integrin β1. These findings suggest that TFEB might represent a novel target for the pharmacological control of pathological angiogenesis and bring new insights in the mechanism sustaining TFEB control of endocytosis.

**Supplementary Information:**

The online version contains supplementary material available at 10.1007/s10456-022-09840-x.

## Introduction

The plasma membrane of endothelial cells (ECs) is a very dynamic structure characterized by the presence of different adhesion receptors that regulate interconnected signaling pathways leading to blood vessel development and angiogenic remodeling [1, 2]. Among these receptors, integrins are αβ heterodimeric transmembrane protein dimers connecting the extracellular matrix (ECM) to the actin cytoskeleton and playing strategic roles during physiological and pathological angiogenesis [3, 4]. In ECs, integrins containing the β1 subunit are major ECM receptors that control cell adhesion, motility, proliferation and apoptosis [3, 4]. On the surface of ECs, integrins exist in a dynamic equilibrium between a bent/closed (inactive) and an extended/open (active) conformation, which exhibit low and high affinity for ECM ligands, respectively [5, 6].

The dynamics of ECM adhesions and the modulation of their strength depend not only on the conformational activation of integrins, but also on their localization in specific plasma membrane microdomains and endo-exocytic trafficking [6]. Depending on their subunit composition and ligand specificity, integrins can be internalized through clathrin-dependent or clathrin-independent pathways [6]. Caveolae are abundant surface pits formed by the assembly of cytoplasmic proteins on a platform generated by the plasma membrane embedded proteins caveolin-1 (CAV-1) and cavin and by membrane lipids [7]. The sequestration of high amounts of cholesterol is essential for caveolae formation, stability, and function [7]. Indeed, cholesterol is necessary for the aggregation of 8S CAV-1 oligomers into 70S CAV-1 multimers and for the insertion of hydrophobic central and C-terminal palmitoylated regions of CAV-1, within the plasma membrane [8]. Reducing cholesterol disrupts the integrity of caveolae, which results in its collapse into the plane of the plasma membrane and an uneven diffusion of CAV-1 [9, 10]. β1 subunit-containing integrins undergo caveolar endocytosis after binding to different ligands, including fibronectin, type I collagen, and glycosphingolipids [11]. In ECs, loss-of-function strategies targeting CAV-1 result in changes in the cellular shape, impairment of β1 integrin endocytosis, cell cycle G1/S phase transition, migration, and angiogenesis [12, 13]. Furthermore, in ECs, cholesterol regulates focal adhesion (FA) formation [14] and the organization of the actin cytoskeleton [15].

Transcription factor EB (TFEB), which was originally described as an oncogene involved in the onset of a subset of juvenile renal carcinomas [16], plays a major role as a regulator of lysosome biogenesis, autophagy, and metabolism [16–22]. This molecule is characterized by a basic helix-loop-helix leucine zipper structure, and its binding to DNA is mediated by an E-box nucleotide motif (GTCACGTGAC) named the “coordinated lysosomal expression and regulation” (CLEAR) motif [19, 20]. Under nutrient-rich conditions, TFEB is cytosolic and largely inactive, and the mTORC1-mediated phosphorylation of TFEB Ser142 and 211 serves as a docking site for chaperone 14-3-3, which sequesters it in an inactive state in the cytosol and favors its degradation. When cells sense nutrient deficiency and mTORC1 is inactive, TFEB moves to the nucleus, where it binds to the CLEAR motif in the promoter region of target genes [19–24]. We and others have revealed that TFEB is an important regulator of blood vessel formation and function [25–29]. Similar to its role in other cell types and organs [16–24], TFEB modulates genetic programs in vascular cells governing membrane traffic, lysosome biogenesis, autophagy, and exocytosis [25–29]. Furthermore, by coordinating the expression of genes involved in the control of the cell cycle and the trafficking of VEGF receptor 2 (VEGFR2), TFEB regulates EC proliferation [25]. Indeed, we previously showed that TFEB silencing leads to VEGFR2 accumulation at the plasma membrane, which is due to the impairment of its endocytosis through CAV-1-rich membrane rafts, a mechanism known to tune the signaling activities of VEGFR2 [30, 31], and its reduced degradation via the lysosomal autophagic pathway.

Here, we identify a novel function of TFEB that controls EC adhesion to the ECM by regulating the formation and turnover of adhesion sites via the expression of genes sustaining a metabolic program. By promoting the expression of genes involved in cholesterol synthesis and modulating the CAV-1-mediated internalization of β1 integrins, TFEB plays a key role in the control FA dynamics in ECs.

## Results

### TFEB inhibits EC adhesion and FA formation

**Fig. 1:**
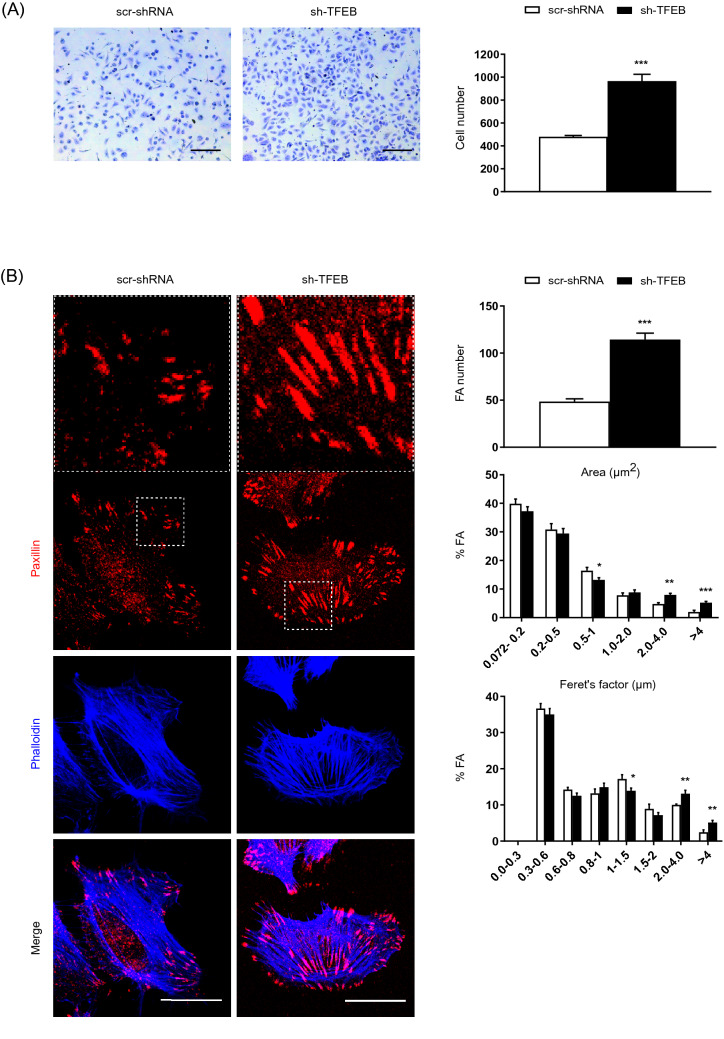
Modulation of FAs and adhesion by the silencing of physiological TFEB. **A** Representative images of adherent scr-shRNA- and sh-TFEB-ECs seeded on FN (10 × magnification, scale bar: 200 µm). The numbers of adherent cells are plotted as the mean ± SEM (*n* = 3 independent experiments; ***p < 0.0001 for sh-TFEB-ECs *versus* scr-shRNA-ECs, as determined by Student’s *t* test). **B** Confocal microscopy analysis of scr-shRNA- and sh-TFEB-ECs stained with anti-paxillin Ab and phalloidin-647 (scale bar: 25 µm). The bar graphs show the quantification of the FA number, the FA area distribution (as the % of FAs with a definite area range *versus* the total number of FAs for each cell) and the Feret’s diameter distribution (as the % of FAs characterized with a definite range of Feret’s diameter *versus* the total number of FAs for each cell) (*n* = 20 cells per condition pooled from three independent experiments; values as mean ± SEM; ***p < 0.0001, **p < 0.001, and *p < 0.01 for sh-TFEB-ECs *versus* scr-shRNA-ECs, as determined by Student’s *t *test)

To understand the physiological role of TFEB in the regulation of EC adhesion, we exploited cells in which endogenous TFEB was silenced by specific sh-RNA (sh-TFEB-ECs) and compared them with ECs carrying a control short hairpin RNA (scr-shRNA-ECs) (Fig. S1 A and B). ECs were seeded on fibronectin (FN), a prototypic provisional ECM component that is crucial for the formation of new blood vessels [32] and the number of adherent cells was quantified (Fig. 1A and Fig. S1 C). The adhesion of sh-TFEB-ECs was increased compared with that of scr-shRNA-ECs (Fig. 1A and Fig. S1 C) thus suggesting that TFEB may modulate angiogenesis by influencing not only EC proliferation [25], but also EC adhesion to the ECM.

To directly investigate this hypothesis, we analyzed the pattern of F-actin and paxillin-labeled FAs in control and TFEB-silenced ECs plated on FN, by confocal fluorescence microscopy. As shown in Fig. 1B and Fig. S1 D, both sh-TFEB-ECs and scr-shRNA-ECs displayed typical elongated and thick actin stress fibers, and TFEB silencing resulted in the formation of more numerous and larger FAs, as evaluated by measuring the area and Feret’s diameter (*i.e.,* the longest FA axis) (Fig. 1B and Fig. S1 D). Indeed, in TFEB-silenced ECs FA area and Feret’s diameter were 60% and 50% higher than in control ECs, respectively (Average FA area: scr-shRNA-ECs 1.2 ± 0.04 µm^2^ and sh-TFEB-ECs 1.9 ± 0.05 µm^2^, *p* = 0.005; average Feret’s diameter: scr-shRNA-ECs 1.4 ± 0.03 µm and sh-TFEB-ECs 2.1 ± 0.04 µm, *p* = 0.003; values as means ± SEMs; p as determined by Student’s *t *test; *n* = 20 cells per condition pooled from 3 independent experiments) (Fig. 1B).

Hence, the observed increased adhesion of TFEB-silenced ECs to provisional ECM proteins might rely on defects in FA turnover and dynamics.

### TFEB silencing increases surface β1 integrins, fibrillar adhesions and fibronectin fibrillogenesis

Integrins are key ECM receptors and their endo-exocytic trafficking is crucial for the regulation of FA turnover and ECs adhesion [6]. In particular, α5β1 integrin recognizes FN as specific ECM ligand supporting its remodeling, internalization and degradation [32–34].

**Fig. 2:**
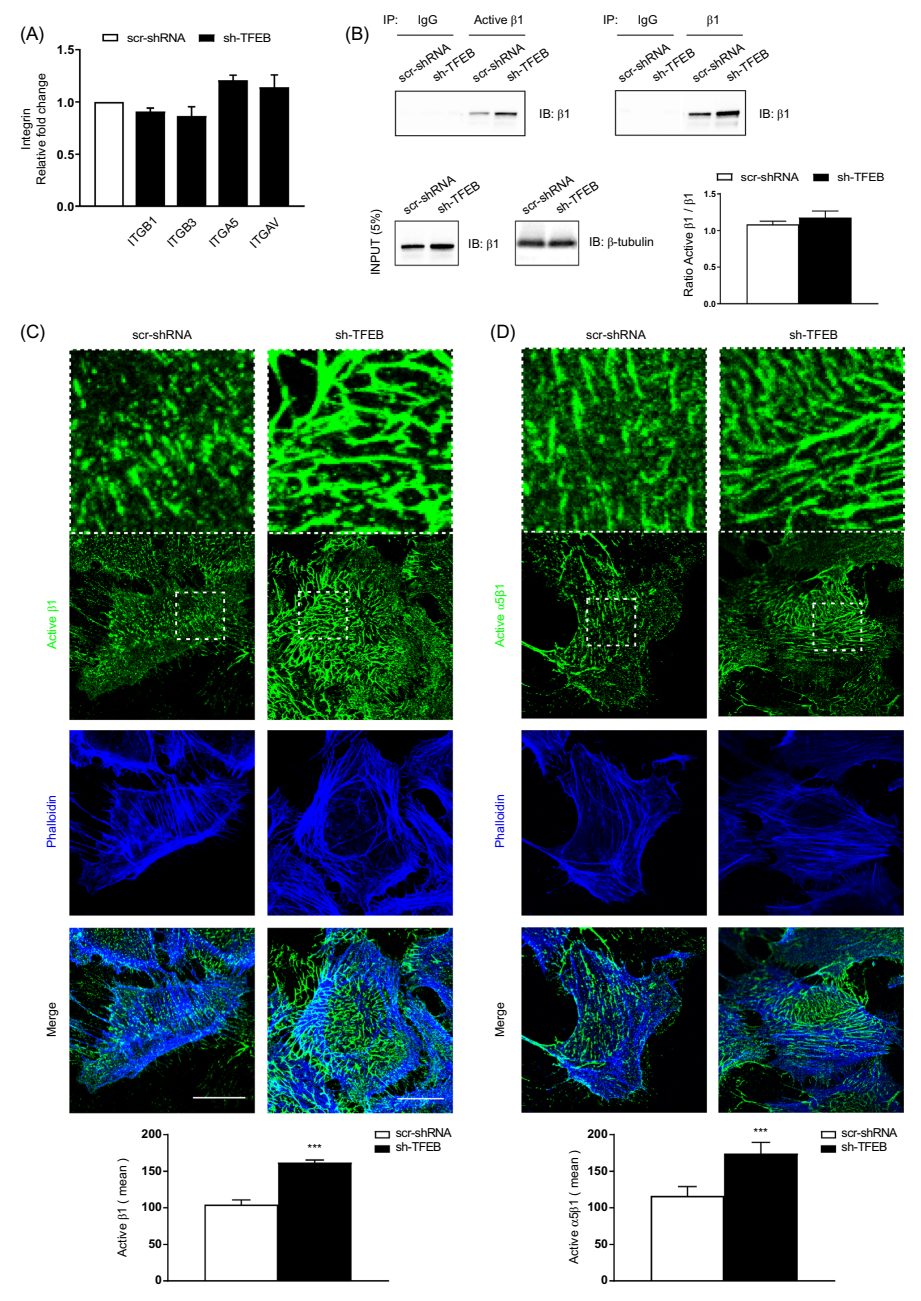
TFEB silencing increases the integrin protein levels. **A** qPCR of *ITGB1, ITGB3, ITGA5 and ITGAV* expression in scr-shRNA and sh-TFEB-ECs. Data are expressed as relative fold change in sh-TFEB-ECs compared with the expression in scr-shRNA-ECs after normalization to the housekeeping gene *TBP* (*n* = 3 independent experiments, values as mean ± SEM; *p* = ns sh-TFEB-ECs *versus* scr-shRNA-ECs by Student’s *t *test). **B** Representative western blot of active and total β1 integrins immunoprecipitated from scr-shRNA- or sh-TFEB-ECs lysates. After immunoprecipitation (IP) with anti-active β1 integrin (9EG7) and anti-total β1 integrin antibodies, proteins were blotted (IB) with anti-total β1 integrin antibody. In the same lysates used for immunoprecipitation, the endogenous expression of total β1 integrin and β-tubulin were blotted with the specific antibodies and presented as “input”. **C**, **D** Confocal microscopy analysis of plasma membrane active-β1 and active- α5β1 integrin expression and localization in living scr-shRNA- and sh-TFEB-ECs following incubation with anti-active β1 integrin (9EG7), active-α5β1 (SNAKA-51) and phalloidin-647 (scale bar: 25 µm). The bar graphs show the quantification of the mean intensity of active-β1 integrin and active- α5β1 (*n* = 10 cells per condition pooled from three independent experiments; values as mean ± SEM; ***p < 0.0001 sh-TFEB-ECs *versus* scr-shRNA-ECs, as determined by Student’s *t *test)

Although TFEB silencing in ECs did not modify the transcription of the major integrin subunit genes, such as *ITGA5*, *ITGAV*, *ITGB1* and *ITGB3* (Fig. 2A and Fig. S2 A), it increased the cellular amount of total and conformationally active β1 and active α5β1 integrin (Fig. S1 B, Fig. 2B and Fig. S2 B). The unchanged ratio between active/total β1 in sh-TFEB- compared to scr-shRNA-ECs suggested that the lack of TFEB increases total β1 integrin amounts, but not its activation (Fig. S1 B, Fig. 2B and Fig. S2 B). In addition, we evaluated the distribution of conformationally active β1 and α5β1 integrin at the plasma membrane. Confocal immunofluorescence microscopy showed that, compared to scr-shRNA-ECs, sh-TFEB-ECs were characterized by higher amount of active β1 and α5β1 integrin on the cell surface (Average fibrillar adhesion size: active β1^+^: scr-shRNA-ECs 2.6 ± 0.6 µm; sh-TFEB-ECs 10.9 ± 2.0 µm; active α5β1^+^: scr-shRNA-ECs 3.8 ± 1.1 µm; sh-TFEB-ECs 12.3 ± 3.4 µm; values as mean ± SEM, p < 0.0001 as determined by Student’s *t *test; *n* = 10 cells from 3 independent experiments) (Fig. 2C and D).

Compared to scr-shRNA-ECs, sh-TFEB-ECs displayed not only higher amount and size of FAs but also of fibrillar adhesions, as evidenced by the co-localization of active β1 integrin and the cytoskeletal adaptor tensin (Fig. S1 E).

As previously described [35–37], ECs are able to synthesize endogenous FN dimers that, upon binding to α5β1 integrin, polymerize into an extracellular fibrillary network [11, 34–39]. As expected, ECs physiologically synthesize endogenous FN (Fig. S2 C, Fig. S2 D and Fig. S2 E). Furthermore, TFEB silencing increased both FN quantity and its polymerization into fibrils (Fig. S2 C, Fig. S2 D and Fig. S2 E). Quantitative real time PCR (qPCR), showed that TFEB silencing did not modify FN mRNA level in ECs (Fig. S2 D), suggesting that the observed increase in FN protein level could be probably due to a posttranslational regulation, e.g., endocytosis and degradation.

Integrin-mediated adhesion to the ECM activates the autophosphorylation of focal adhesion kinase (FAK) and Src family kinases along with the recruitment of adaptor proteins [40–42]. We found that both FAK and Src proteins were more activated in sh-TFEB- vs scr-shRNA-ECs, as evidenced by the Western blot analysis of the pTyr397/total FAK and pTyr416/Total Src protein ratio (Fig. S2 F).

### TFEB silencing inhibits β1 integrin internalization and degradation

**Fig. 3:**
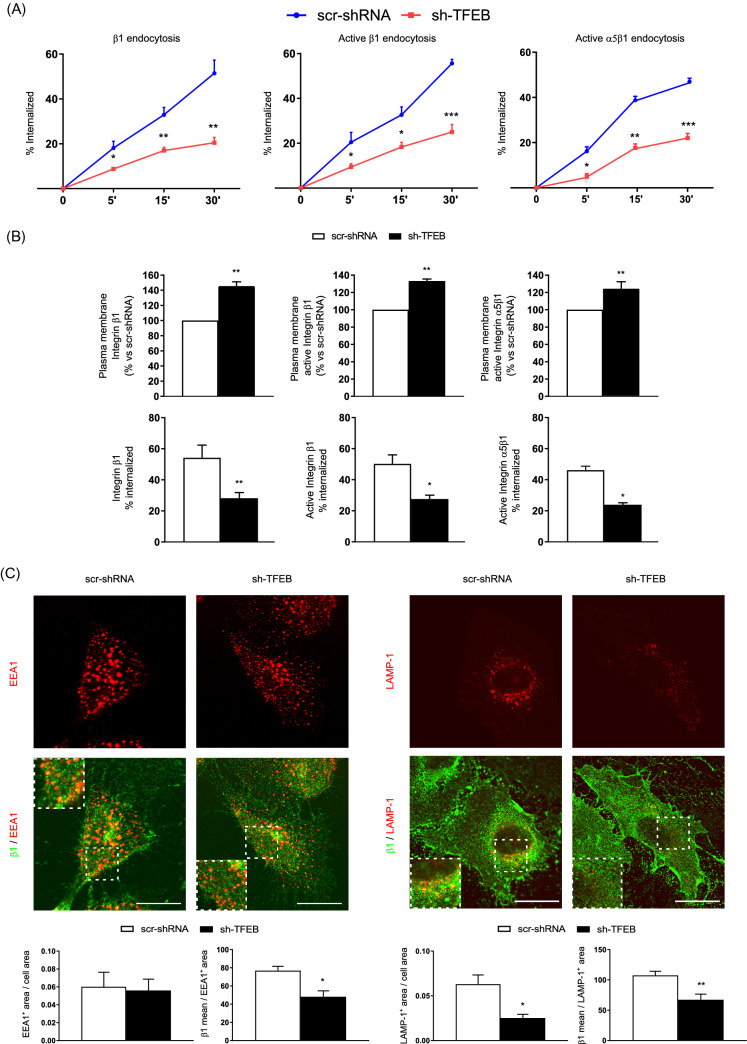
TFEB silencing inhibits β1 integrin internalization. **A** Time-course analysis of the relative amounts of internalized total and active β1 and active- α5β1 integrin in scr-shRNA-ECs and sh-TFEB-ECs. Integrin internalization was evaluated by integrin internalization assay and capture ELISA assay (*n* = 3 independent experiments, values as mean ± SEM, ***p < 0.0001, **p < 0.001, *p < 0.01 all samples *versus* scr-shRNA-ECs as determined by Student’s *t *test). **B** Analysis of the relative amounts of internalized total and active β1 and active-α5β1 integrin in scr-shRNA-ECs and sh-TFEB-ECs (*n* = 3 independent experiments, values as mean ± SEM, **p < 0.001, *p < 0.01 all samples *versus* scr-shRNA-ECs by Student’s *t *test). **C** Confocal microscopy analysis of total β1 integrin localization in EEA1^+^ and LAMP-1^+^ vesicles in scr-shRNA-ECs and sh-TFEB-ECs stained with anti-β1 integrin, anti-EEA1 and anti-LAMP-1 Abs (scale bar: 50 µm). Bar graph shows the quantification of EEA1^+^ and LAMP-1^+^ areas per cell area and the amount of β1 integrin accumulated in EEA1^+^ and LAMP-1^+^ areas (*n* = 20 cells per condition pooled from three independent experiments, values are mean ± SEM; *p < 0.01, **p < 0.001 scr-shRNA- *versus* sh-TFEB-ECs as determined by Student’s *t *test)

Because we previously demonstrated that TFEB regulates the trafficking of transmembrane protein receptors [25], we wondered whether the increase of surface β1 integrins, fibrillar adhesions, and fibronectin fibrillogenesis observed in TFEB-silenced ECs could be due to alterations in β1 integrin internalization. Biochemical time course analyses showed that, compared to controls, TFEB silencing decreased the internalization of both total and active β1 and active α5β1 integrins (41.4 ± 7.7%, 45.0 ± 5.8%, 46.9 ± 8.1%, after 30 min, respectively; values as mean ± SEM, *p* = 0.001 and *p* = 0.0003, respectively as determined by Student’s *t-*test; n = 3 independent experiments) (Fig. 3A and B). Of note, in sh-TFEB-ECs, the significantly reduced endocytic rate was paralleled by the simultaneous increase of both total and active β1 and active α5β1 integrin distribution at the cell surface (Fig. 3B).

Upon internalization, integrins are delivered to early endosomes and then either routed to lysosomes for degradation or recycled back to the plasma membrane [5, 6, 43–45]. TFEB regulates via CLEAR motif the transcription of genes encoding for vesicle membrane proteins, hydrolases and proteins involved in the regulation of endocytosis and exocytosis processes [18–29]. The lack of TFEB resulted in the reduction of LAMP-1^+^ lysosomes (64.6 ± 6,.9%), but not EEA1^+^ early endosomes (Fig. 3C) and, as evidenced in Fig. 3C and Fig. S3 A, total and active β1 and active α5β1 integrin amounts were significantly reduced in both early endosomes and lysosomes in agreement with the observed reduction of integrin internalization (Fig. 3A and B). Moreover, TFEB silencing resulted in the reduced degradation rate of internalized active β1 integrin (Fig. S3 B).

### TFEB silencing reduces CAV-1 plasma membrane localization and caveolar β1 integrin internalization

**Fig. 4:**
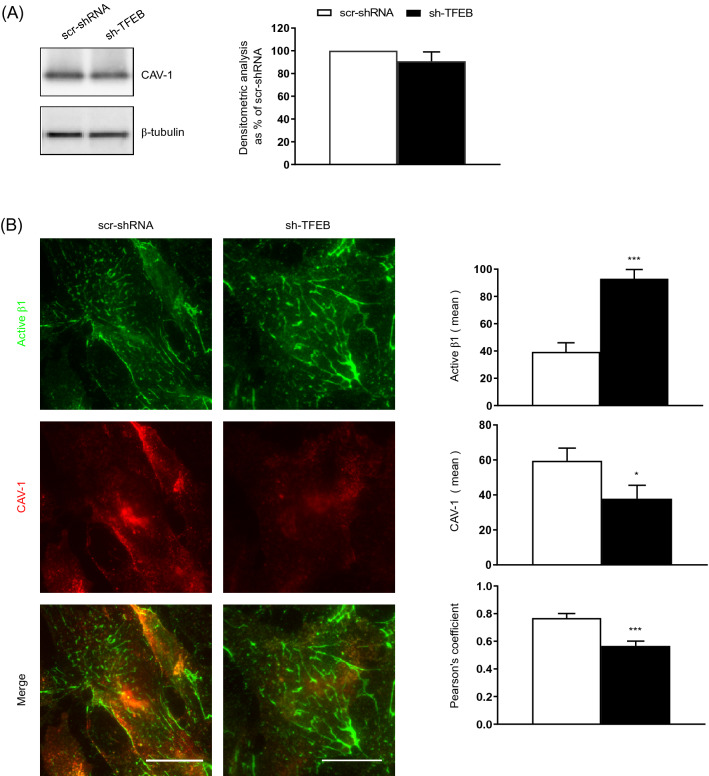
TFEB silencing inhibits CAV-1 membrane distribution and integrin/CAV-1 proximity. **A** Representative western blots of cellular amount of CAV-1 expression in scr-shRNA- and sh-TFEB-ECs. Bar graph shows the densitometric analysis expressed as the ratio between CAV-1 and β-tubulin (*n* = 3 independent experiments, values as mean ± SEM; *p* = ns sh-TFEB- *versus* scr-shRNA-ECs by Student’s *t *test). **B** Representative TIRF images of active β1 integrin and CAV-1 in living scr-shRNA- and sh-TFEB-ECs. After in vivo incubation with anti-active β1 integrin (9EG7) antibody, cells were stained with anti-CAV-1 antibody (scale bar: 25 µm). The bar graphs show the quantification of the mean intensity of active β1 integrin and CAV-1 and the proximity of the two proteins analyzed with Pearson’s coefficient (*n* = 20 cells per condition pooled from three independent experiments; values as mean ± SEM; ***p < 0.0001 and *p < 0.01 for sh-TFEB-ECs *versus* scr-shRNA-ECs, as determined by Student’s *t *test)

**Fig. 5:**
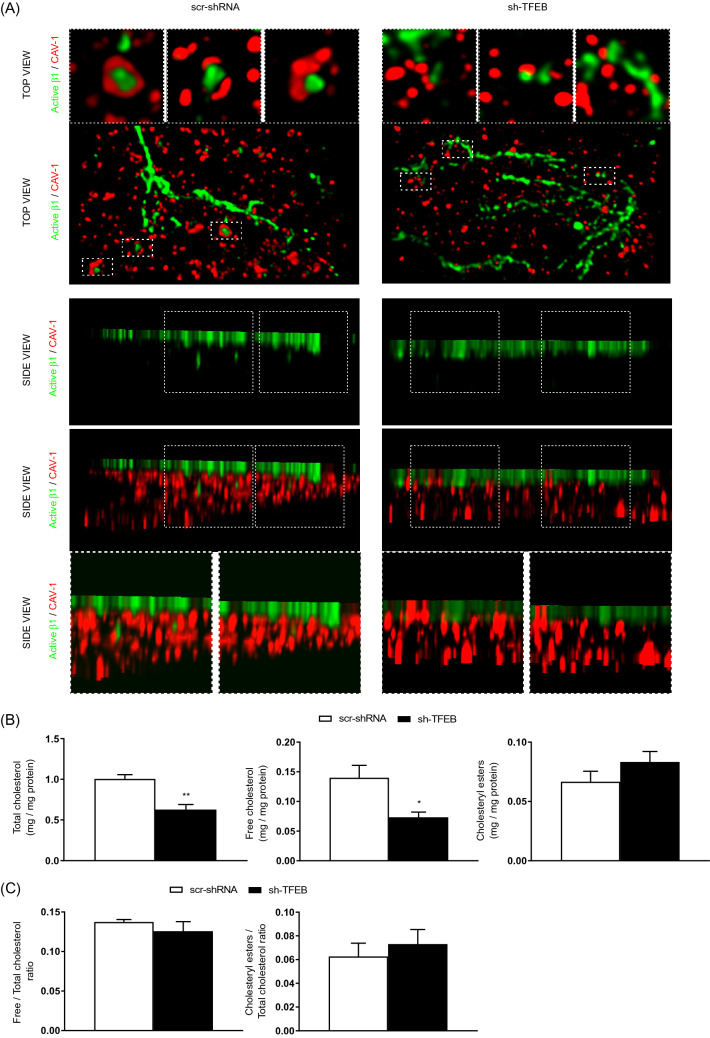
TFEB silencing reduces integrin/CAV-1 proximity and integrin internalization via cholesterol synthesis inhibition. **A** Representative 3D-STED super-resolution image of total/ active β1 integrin and CAV-1 expression and localization in living scr-shRNA- and sh-TFEB-ECs. After in vivo incubation with anti-active β1 integrin (9EG7) antibody, cells were stained with CAV-1 antibody (scale bar: 0.5 µm; *n* = 5 cells per condition pooled from three independent experiments). **B** The bar graphs show the quantification of total cholesterol, free cholesterol and cholesteryl esters in scr-shRNA- and sh-TFEB-ECs (*n* = 3 independent experiments; values as mean ± SEM; **p < 0.001 and *p < 0.01 for sh-TFEB-ECs *versus* scr-shRNA-ECs, as determined by Student’s *t *test). **C**The bar graphs show the ratio between free and total cholesterol and cholesteryl esters and total cholesterol in scr-shRNA- and sh-TFEB-ECs (*n* = 3 independent experiments; values as mean ± SEM; p = ns for sh-TFEB-ECs *versus* scr-shRNA-ECs, as determined by Student’s *t *test)

CAV-1 participates in the regulation of β1 integrin endocytosis [7]. We previously revealed that the silencing of TFEB in ECs dysregulates the interplay between CAV-1 and VEGFR2 [25]. Hence, we posited that the decreased β1 integrin internalization observed in sh-TFEB-ECs could be due to alterations in CAV-1-dependent internalization and trafficking. In ECs, TFEB silencing did not change *CAV-1* gene transcription (The relative fold change in sh-TFEB-ECs compared with the expression in scr-shRNA-ECs after normalization to the housekeeping gene *TBP* was 0.9 ± 0.1; value as mean ± SEM; *p* = ns as determined by Student’s *t *test; *n* = 3 independent experiments) and cellular protein levels compared to control cells (Fig. 4A). However, total internal reflection fluorescence (TIRF) microscopy analysis of the EC surface showed a reduction of CAV-1 at plasma membrane (Fig. 4B) and a decreased proximity between CAV-1 and active β1 integrin (Fig. 4B) in sh-TFEB-ECs respect of scr-shRNA-ECs. Stimulated emission depletion (STED) confocal fluorescence microscopy analysis and 3D‐STED super‐resolution imaging revealed that CAV-1 and active β1 integrin were in close proximity and co-internalized in scr-shRNA-ECs but not in sh-TFEB-ECs (Fig. 5A).

### TFEB modulates integrin internalization through cholesterol homeostasis

To further analyze the role of TFEB in CAV-1-mediated β1 integrin endocytosis, we subsequently assessed whether TFEB could regulate the homeostasis of cholesterol, which is instrumental in CAV-1 patterning at the plasma membrane [7–15]. Biochemical analysis of ECs demonstrated a clear reduction in the total cellular cholesterol quantity after TFEB silencing (Fig. 5B). In scr-shRNA- and sh-TFEB-ECs we also quantified the amounts of membrane-free and unesterified cholesterol, which is mainly contained in the plasma membrane [46], and esterified cholesterol, which is typically contained in cytosolic stores. Interestingly, the loss of TFEB reduced free and esters cholesterol per se (Fig. 5B), but it did not modify the ratio between free/total cholesterol and esters/total cholesterol (Fig. 5C), suggesting a defect in cholesterol synthesis, but not an alteration of its subcellular distribution.

**Fig. 6:**
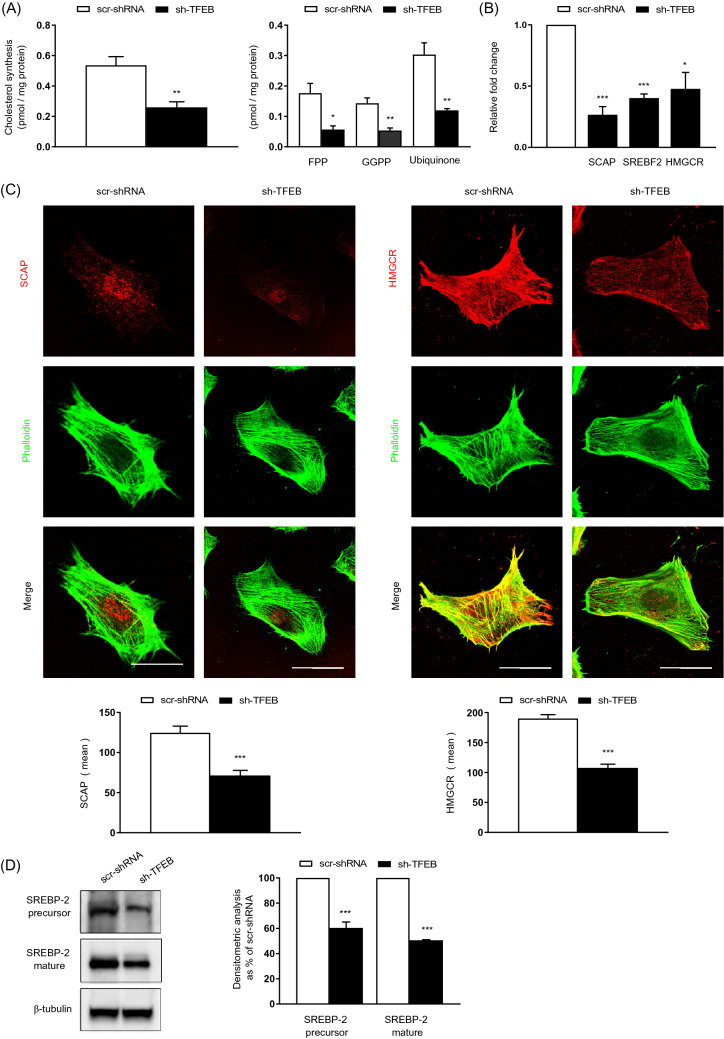
TFEB silencing inhibits cholesterol synthesis. **A** The bar graphs show the quantification of de novo cholesterol, FPP, GGPP and ubiquinone synthesis in scr-shRNA- and sh-TFEB-ECs grown in medium containing [3H] acetate (*n* = 3 independent experiments; values as mean ± SEM; ***p < 0.0001, **p < 0.001 and *p < 0.01 for sh-TFEB-ECs versus scr-shRNA-ECs, as determined by Student’s *t* test). **B** qPCR of *SCAP, SREBF-2* and *HMGCR* expression in scr-shRNA- and sh-TFEB-ECs. The data are expressed as the relative fold changes in sh-TFEB-ECs compared with the expression in scr-shRNA-ECs after normalization to the housekeeping gene *TBP* (*n* = 3 independent experiments; values as mean ± SEM; ***p < 0.0001 and *p < 0.01 for sh-TFEB-ECs *versus* scr-shRNA-ECs, as determined by Student’s *t *test). **C** Confocal microscopy analysis of SCAP and HMGCR expression in scr-shRNA- and sh-TFEB-ECs stained with anti-SCAP or anti-HMGCR antibodies and phalloidin-488 (scale bar: 25 µm). The bar graph shows the quantification of the mean intensity of SCAP or HMGCR (*n* = 20 cells per condition pooled from three independent experiments; values as mean ± SEM; ***p < 0.0001 for sh-TFEB-ECs *versus* scr-shRNA-ECs, as determined by Student’s *t *test). **D** Representative western blots of the cellular amounts of unprocessed or mature SREBP2 expression in scr-shRNA- and sh-TFEB-ECs. The bar graph shows the densitometric results expressed as the ratio of SREBP2 to β-tubulin (*n* = 3 independent experiments; mean ± SEM; ***p < 0.0001 for sh-TFEB-ECs *versus* scr-shRNA-ECs, as determined by Student’s *t *test)

The synthesis of cholesterol and cholesterol intermediates and side products, such as the isoprenoids farnesyl pyrophosphate (FPP), geranylgeranyl pyrophosphate (GGPP) and the isoprenoid-containing molecule ubiquinone, was clearly reduced in sh-TFEB-ECs, compared with scr-shRNA-ECs (Fig. 6A). In contrast, the lack of TFEB did not affect cholesterol uptake and efflux (Cholesterol uptake: sh-TFEB-ECs as % of scr-shRNA-ECs 101.1 ± 10.1; cholesterol efflux: sh-TFEB-ECs as % of scr-shRNA-ECs 96.7 ± 6.7; values as mean ± SEM, *p* = ns as determined by Student’s *t *test; *n* = 3 independent experiments).

The biosynthesis of cholesterol is regulated by different enzymes and the endoplasmic reticulum (ER) transmembrane protein complex sterol-regulatory element-binding protein-2 (SREBP-2, *SREBF-2*)/SREBP cleavage-activating protein (SCAP) [46]. The N-terminal domain of membrane-associated SREBP-2 displays a basic helix-loop-helix (bHLH) folding. Upon SCAP-mediated trafficking from the ER to the Golgi, SREBP-2 is cleaved by proteases to release its bHLH in the cytosol that is then translocated into the nucleus to act as a transcription factor. The bHLH domain of SREBP2 induces the transcription of key genes involved in cholesterol metabolism, including β-Hydroxy β-methylglutaryl-CoA reductase (HMGCR), the rate limiting enzyme of cholesterol synthesis [46].

Remarkably, we found that TFEB silencing inhibited the transcription of the *SREBF-2* and *SCAP* genes and the SREBP-2 target gene *HMGCR* (Fig. 6B). Furthermore, as evidenced by confocal fluorescence microscopy and immunoblot analysis (Fig. 6C and D), and consistent with the significant decrease in the biosynthesis of cholesterol and its intermediates (Fig. 6A), the levels of SCAP protein, HMGCR protein (Fig. 6C), unprocessed SREBP-2 precursor protein, and cleaved mature N-terminal bHLH domain of SREBP-2 (Fig. 6D) were significantly reduced in sh-TFEB-ECs. After TFEB silencing HMGCR expression and cholesterol synthesis were similar to that observed after HMGCR silencing in scr-shRNA-ECs, suggesting that indeed TFEB may act by promoting the transcription of *HMGCR* (Fig. S4 A). Even after HMGCR silencing in scr-shRNA-ECs the ratio between free/total cholesterol and cholesteryl esters/total cholesterol were unchanged (Fig. S4 A).

Altogether, these data suggest that in ECs lacking TFEB the surface accumulation of β1 integrins and the ensuing enlargement and stabilization of FAs may be due to the reduction of plasma membrane-free cholesterol, CAV-1 clustering, and caveolae-mediated β1 integrin internalization [10–15].

**Fig. 7:**
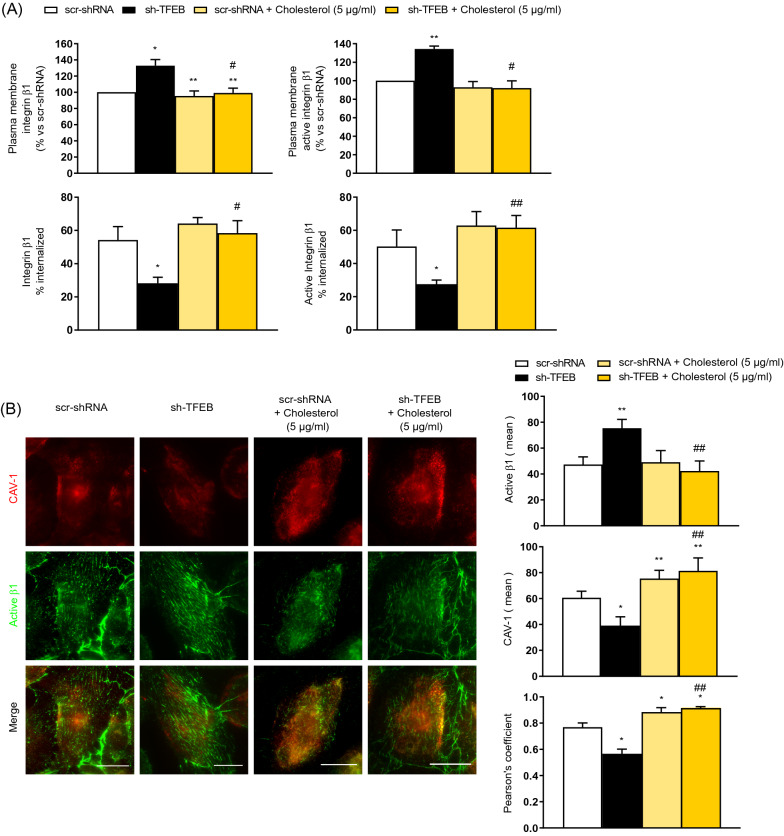
Exogenous cholesterol abrogates TFEB silencing effects on integrin and CAV-1 membrane distribution. **A** Analysis of the relative amounts of internalized total and active β1 integrin in scr-shRNA-ECs and sh-TFEB-ECs supplemented or not supplemented with cholesterol (5 µg/ml, 24 h). Integrin internalization (after 30 min of induction) was evaluated by integrin internalization assay and capture ELISA assay (*n* = 3 independent experiments, values as mean ± SEM, **p < 0.001, *p < 0.01 all samples *versus* scr-shRNA-ECs by Student’s *t *test; ^##^*p* < 0.001 and ^#^*p* < 0.01 for sh-TFEB-ECs supplemented with cholesterol *versus* sh-TFEB-ECs, as determined by Student’s *t *test). **B** Representative TIRF images of active β1 integrin and CAV-1 in living scr-shRNA- and sh-TFEB-ECs supplemented or not supplemented with cholesterol (5 µg/ml, 24 h). After in vivo incubation with anti-active β1 integrin (9EG7) antibody, cells were stained with anti-CAV-1 antibody (scale bar: 25 µm). The bar graphs show the quantification of the mean intensity of active β1 integrin and CAV-1 and the proximity of the two proteins analyzed with Pearson’s coefficient (*n* = 20 cells per condition pooled from three independent experiments; values as mean ± SEM; **p < 0.001 and *p < 0.01 for all samples *versus* scr-shRNA-ECs, as determined by Student’s *t-*test; ^##^*p* < 0.001 for sh-TFEB-ECs supplemented with cholesterol *versus* sh-TFEB-ECs, as determined by Student’s *t-*test).

**Fig. 8:**
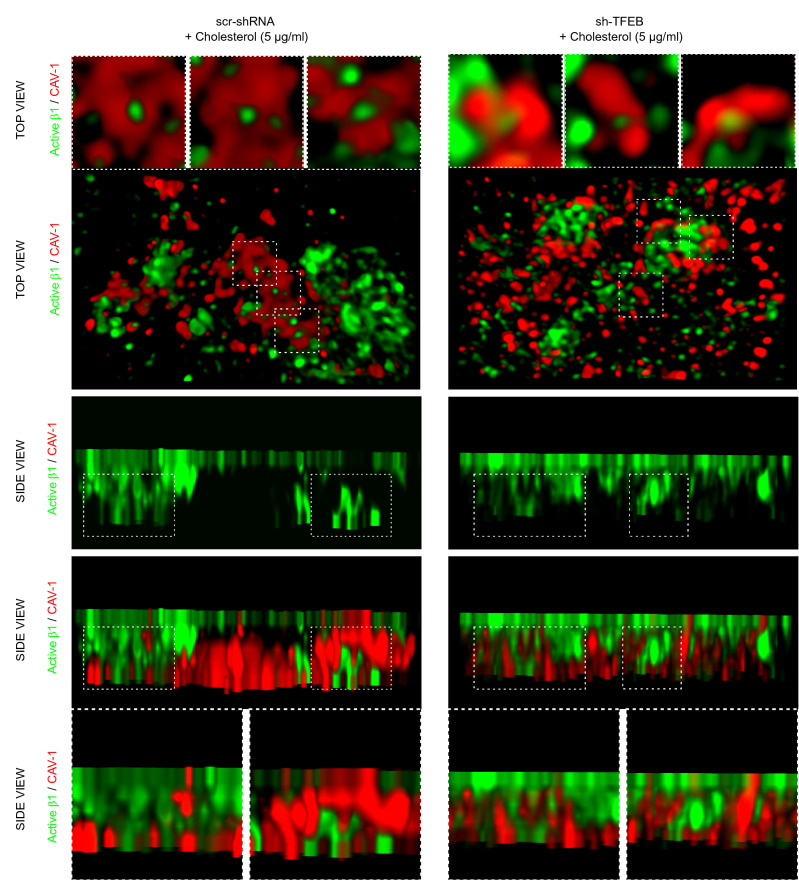
Exogenous cholesterol rescues TFEB silencing inhibition of integrin internalization. Representative 3D-STED super-resolution image of active β1 integrin and CAV-1 expression and localization in living scr-shRNA- and sh-TFEB-ECs supplemented with cholesterol (5 µg/ml, 24 h). After in vivo incubation with anti-active β1 integrin (9EG7) antibody, cells were stained with anti-CAV-1 antibody (scale bar: 0.5 µm; *n* = 5 cells per condition pooled from three independent experiments)

**Fig. 9:**
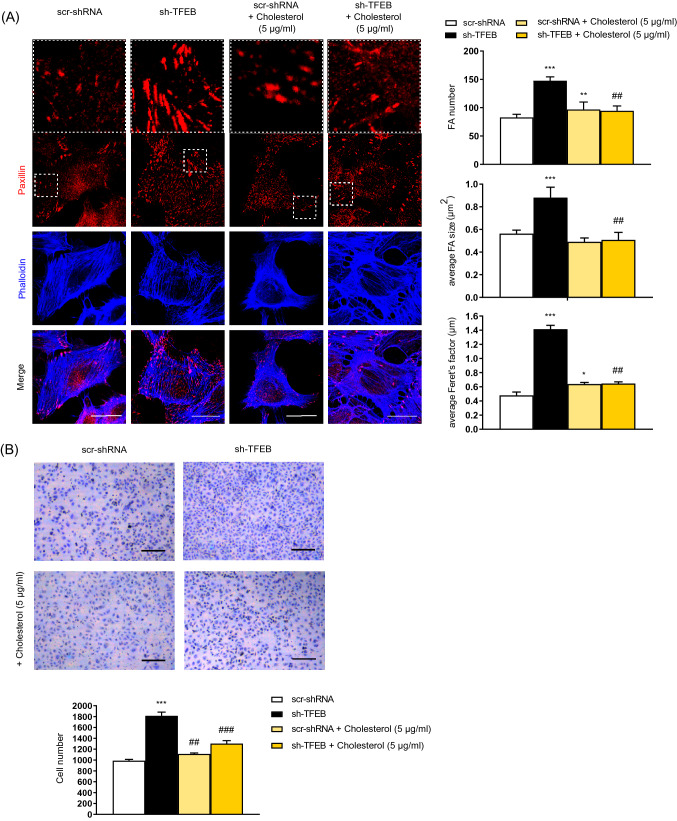
Exogenous cholesterol rescues TFEB silencing effects on FAs development and ECs adhesion. **A** Confocal microscopy analysis of scr-shRNA- and sh-TFEB-ECs supplemented or not supplemented with cholesterol (5 µg/ml, 24 h) stained with anti-paxillin Ab and phalloidin-647 (scale bar: 25 µm). The bar graphs show the quantification of the FA number, the average FA area and the average Feret’s diameter (*n* = 20 cells per condition pooled from three independent experiments; values as mean ± SEM; ***p < 0.0001, **p < 0.001 and *p < 0.001 for all samples *versus* scr-shRNA-ECs, as determined by Student’s *t *test; ^##^*p* < 0.001 for sh-TFEB-ECs supplemented with cholesterol *versus* sh-TFEB-ECs, as determined by Student’s *t *test). **B** Representative images of adherent scr-shRNA- and sh-TFEB-ECs supplemented or not supplemented with cholesterol (5 µg/ml, 24 h) seeded on FN (10 × magnification, scale bar: 200 µm). The numbers of adherent cells are plotted as the mean ± SEM (*n* = 3 independent experiments; ***p < 0.0001 for sh-TFEB-ECs *versus* scr-shRNA-ECs, as determined by Student’s *t *test; ^###^*p* < 0.0001 and ^##^*p* < 0.001 for sh-TFEB-ECs supplemented with cholesterol *versus* sh-TFEB-ECs, as determined by Student’s *t* test)

To validate these assumptions, we performed experiments in which cholesterol amounts were modulated (Figs. 7, 8, 9 and Fig. S4 C-D). In particular, we depleted endogenous cholesterol by treating scr-shRNA- and sh-TFEB-ECs with β-methyl cyclodextrin (βMCD) (5 mM, 1 h). Similarly to what observed in sh-TFEB-ECs, the depletion of endogenous cholesterol in scr-shRNA- ECs reduced the internalization of both total and active β1 integrin (Fig. S4 B). Moreover, cholesterol depletion in scr-shRNA-ECs increased both total and active β1 integrin amounts albeit to a lesser extent than in sh-TFEB ECs (Fig. S4 C). As expected, treatment of scr-shRNA-ECs with the specific inhibitor of lysosome activity, the vacuolar H^+^ ATPase inhibitor bafilomycin A1 [47] also due to the increase of total and active β1 integrins amount (Fig. S4 C) but only the contemporary presence of bafilomycin A1 and cholesterol depletion mimicked the effect on total and active β1 integrin observed in sh-TFEB-ECs (Fig. S4 C).

On the contrary, we tried to rescue sh-TFEB-ECs phenotype by ECs treatment with exogenous cholesterol. Upon depletion of endogenous cholesterol by βMCD (5 mM, 1 h) and treatment with different concentrations of exogenous cholesterol (1–30 µg/ml, 24 h), we found that the minimal concentration of exogenous cholesterol capable of restoring in sh-TFEB-ECs total cellular cholesterol levels comparable to those of scr-shRNA-ECs was 5 µg/ml, a concentration that we hence employed in rescue experiments (Fig. S4 D and Figs. 7, 8, 9). In sh-TFEB-ECs the normalization of total cholesterol quantity led to the rescue of free cholesterol level (Fig. S4 D). Free/total and cholesteryl esters/total cholesterol ratios were similar after adding 5 µg/ml of exogenous cholesterol in the two cell types (Ratio free / total cholesterol: scr-shRNA-ECs + cholesterol 0.14 ± 0.01; sh-TFEB-ECs + cholesterol 0.13 ± 0.1; ratio cholesteryl esters / total cholesterol: scr-shRNA-ECs + cholesterol 0.08 ± 0.01; sh-TFEB-ECs + cholesterol 0.09 ± 0.01; values as mean ± SEM, *p* = ns as determined by Student’s *t *test; *n* = 3 independent experiments). Of note, the addition of exogenous cholesterol in sh-TFEB-ECs restored total and active β1 integrin surface amounts (Fig. 7A and Fig. S5 A) and internalization rates (Fig. 7A) similar to those of scr-shRNA-ECs and substantially, yet not completely, rescued total and active β1 integrin levels (Fig. S4 C), probably because integrin degradation was still blocked (Fig. 3 C, Fig. S3 A and B).

Similarly, TIRF and STED microscopy evidenced that the addition of exogenous cholesterol on sh-TFEB-ECs normalized the distribution of CAV-1 at the plasma membrane, and reestablished the proximity between CAV-1 and active β1 integrin (Fig. 7B and Fig. 8) along with CAV-1-mediated internalization of active β1 integrins (Fig. 8).

Consistently, the addition of exogenous cholesterol rescued abnormal phenotypes observed in sh-TFEB-ECs such as FA average number, size and diameter (Fig. 9A) as well as fibrillar adhesions number and length (Fig. S5 B)(scr-shRNA-ECs + cholesterol: *n* = 158.4 ± 15.4 and average size 0.43 ± 0.04 µm; sh-TFEB-ECs + cholesterol: *n* = 172.1 ± 8 and average size 0.48 ± 0.07 µm; values as means ± SEMs; *p* = ns as determined by Student’s *t *test; *n* = 10 cells per condition pooled from 3 independent experiments) and cell adhesion (Fig. 9B).

## Discussion

In this study, we unveil a novel function of TFEB as a transcriptional regulator of metabolic signaling pathways that control integrin-mediated EC adhesion to the ECM. We reveal that TFEB is involved in the control of cholesterol synthesis via the transcriptional modulation of genes encoding the sterol sensor SCAP and the associated transcription factor and key regulator of cholesterol homeostasis SREBP-2. TFEB-silenced ECs show defects in endogenous cholesterol synthesis and are characterized by inhibition of the cholesterol-dependent clustering of plasma membrane CAV-1, the association β1 integrin with CAV-1 and their internalization. In particular, after TFEB silencing the integrin increased level is mediated by the block of integrin internalization and degradation as the result of the inhibition of cholesterol synthesis, altered distribution of cholesterol on plasma membrane and reduced lysosome-dependent protein degradation.

TFEB is emerging as a key modulator of cellular metabolism, including triglyceride and fatty acid catabolism [48]. Here, we discover that TFEB also promotes endogenous cholesterol synthesis in ECs. Cells sense the endogenous cholesterol levels through the regulatory protein SCAP, which associates with the master regulator of cholesterol biosynthesis SREBP-2 [46]. Cholesterol binding to the sterol-sensing domain of SCAP stabilizes the association of the SCAP/SREBP2 complex with the ER resident protein Insulin Induced Gene 1 (INSIG1). In contrast, in the presence of low endogenous cholesterol levels, the SCAP/SREBP2 complex disconnects from INSIG1 and relocates to the Golgi, where proteases cleave and free the bHLH domain of SREBP2 in the cytosol [46]. The active form of SREBP2 then moves to the nucleus and induces the transcription of cholesterol metabolism genes [46]. Similarly in other cell types [49], TFEB is also able to modulate cholesterol synthesis in ECs. We found that a lack of TFEB resulted in the transcriptional down-regulation of SCAP, SREBP2 and HMGCR. TFEB did not affect cholesterol uptake or efflux, which are two events that might dynamically regulate the amount of intracellular cholesterol, and these findings suggest that reductions in endogenous synthesis might reduce the amount of plasma membrane-associated cholesterol.

Different studies unveiled a cross-talk between the nutrient cellular status and the trafficking of integrins [50–53]. In particular, in cancer cells mTOR inhibition due to nutrient depletion promotes the tensin-dependent maturation of sub-nuclear fibrillar adhesions and the ensuing localized endocytosis of fibronectin-bound active α5β1 integrin [51]. Probably because of the lysosomal degradation of fibronectin and the increased availability of amino acids, the internalization of fibronectin-bound active α5β1 integrin activates mTOR, which in turn impairs the maturation of sub-nuclear fibrillar adhesions [33].

In addition, nutrient and energy sensing crucially relies on AMP-activated protein kinase (AMPK), which is activated by reduced nutrients or increased AMP/ATP ratio [50–54]. Of note, in wild type fibroblasts activated AMPK inhibits the transcription of tensin and in AMPK knockout fibroblasts tensin silencing impairs the activation of α5β1 integrin, cell spreading, traction stress, and fibronectin fibrillogenesis [51, 52].

Nutrient cellular status and mTORC1 activity are strongly involved in TFEB phosphorylation and nuclear translocation [18, 21, 22]. Some studies described an AMPK-mediated activation of TFEB that was abolished in AMPK null models probably via mTORC1 in some cellular conditions [55–57]. Along this line, it is tempting to speculate that the reported inhibitory activity of AMPK on α5β1 integrin function [50–52] may be mediated at least in part by the TFEB-mediated activation of cholesterol and CAV-1-driven β1 integrin endocytosis.

The formation and remodeling of FAs depend on conformational activation, endo-exocytic trafficking and degradation of integrin [5–9, 11–13]. From the plasma membrane, integrins are internalized, *e.g.,* in a clathrin- or caveolin-dependent manner, and then, through endosomal sorting, these molecules are recycled back to the cell surface or degraded in lysosomes [5–9, 11–13].

Caveolae and lipid rafts are specific plasma membrane structures composed by cholesterol, glycosphingolipids, and CAV-1, involved in endocytosis, protein trafficking, and signaling. CAV-1 tightly binds to cholesterol, and both molecules are necessary for the formation of caveolar invaginations and cholesterol-promoting CAV-1 multimerization [7–10]. Various studies have indicated that CAV-1 and cholesterol are key molecules in the regulation of ECs adhesion and migration on FN during angiogenesis [12, 13, 58–62].

Alteration of cholesterol plasma membrane amounts results in the inhibition of key regulators of ECs proliferation such as mTOR and VEGFR2 [59, 61–63]. Reduction of cholesterol trafficking correlates with the inhibition of mTOR activation as a consequence of its translocation on perinuclear lysosomes [61]. Furthermore, in vascular cells cholesterol regulates the receptor membrane topology and activities of VEGFR2, PDGFRβ and Endothelial Activin receptor-like kinase-1 [61–64]. We previously described that endothelial TFEB silencing leads to an anti-angiogenic phenotype supported by VEGFR2 mis-localization at plasma membrane as a consequence of post-transcriptional regulation, de-localization in non-caveolar areas and inhibition of its internalization [25].

Our findings extended the role of TFEB in regulating the functions of membrane receptors and integrins. We propose that under physiological conditions, TFEB participates in integrin traffic by regulating cholesterol bioavailability and CAV-1 subcellular distribution. Indeed, in TFEB-silenced ECs, the plasma membrane patterning of CAV-1, but not its protein levels, was abnormally altered. After TFEB silencing, similarly to previously described via loss-of-function strategies targeting CAV-1 we evidenced an impairment of β1 integrin endocytosis [12, 13]. Furthermore, in ECs, cholesterol regulates FA formation [14] and the organization of the actin cytoskeleton [15].

Endocytosis and degradation or recycling from different vesicular compartments determine the fate of integrins [5, 6, 11, 42–45, 65–72]. In particular, via different mechanisms, active α5β1 integrin after caveolin and clathrin-endocytosis is in part recycled at PM and in part degraded in lysosomes [5, 6, 11, 42–45, 65–72]. FN binding triggers the ubiquitination and the internalization of α5β1 integrin [66]. Most FN-bound α5β1 is de-ubiquitinated and, after FN dissociation, recycled back to plasma membrane [67]. The residual ubiquitinated α5β1 integrin traffics to multivesicular bodies (MVBs) and then to lysosomes where it undergoes degradation [66, 67]. After overexpression of Rab25, endocytosed active α5β1 integrin is also delivered to MVBs from which it recycles back to plasma membrane [68]. Sorting nexin (SNX)-17 binds β1 integrins to prevent their lysosomal degradation and to favor their recycling from endosome to plasma membrane [44, 45]. There are also evidence that internalized FN is actually recycled/secreted from late endosomes and lysosomes [69] and that the endocytosis of FN-bound active α5β1 integrin is sustained by Rab21 [70].

Of note, in ECs was previously described that upon internalization, the FN/active α5β1 integrin complex moves to post Golgi carrier vesicles (PGCs) where α5β1 integrins detach from FN fragments, associates with freshly synthesized FN dimers, and reaches the basolateral side of the plasma membrane [36]. More recently, in endothelium the CDC42-related RHO-family member RHOJ was found to inhibit the accumulation of endocytosed FN-bound/active α5β1 integrins in early endosomes, their transport to PGCs and the ensuing polymerization of new FN [71]. On the contrary, CDC42, which favors PGC-based secretion, opposes RHOJ inhibition of FN fibrillogenesis [71]. In ECs Syntaxin-6 mediates α5β1 integrin recycling while its inhibition sustains the increase of α5 ubiquitination and α5β1 lysosome-dependent degradation [72]. Finally, ECs treatment with macrophage-derived exosomes increases the ubiquitination of integrin β1, its internalization and lysosomal degradation [73].

Interestingly, TFEB regulates the expression of several master genes involved in vesicular trafficking [23–25]. This function supports the observation in pancreatic ductal adenocarcinoma that TFEB promotes the endocytosis of α5β1 integrin and the disassembly focal adhesion by modulating RAB5 and in hepatocellular carcinoma the degradation of β1 integrin by activating lysosome machinery [74, 75].

Altogether these data indicate that TFEB controls integrin endocytosis by a cholesterol-mediate regulation of the caveolin machinery. However, in our experimental conditions we cannot exclude a minor role of clathrin-dependent endocytosis, despite the addition of exogenous cholesterol largely rescued the phenotype changes observed in sh-TFEB-ECs. However, this point requires further investigations. Actually, cholesterol is also involved in clathrin-dependent endocytosis. It has been reported that sterol-dependent membrane properties influence this endocytic mechanism [76], which is blocked by the acute depletion of cholesterol [77].

Our experiments demonstrate that TFEB silencing lead to the increase of ECs adhesion concomitant to a dynamic modification of FAs and FBs supported by elevated amount of integrin on plasma membrane via the regulation of cholesterol synthesis and lysosomal-dependent degradation. Actually, the sh-TFEB-ECs phenotype differs from that observed in other cellular types in which only cholesterol level or integrin recycling was modified while integrin lysosomal degradation was unchanged. In sarcoma cells that express high level of α5β1 integrin the modification of cholesterol level induces changes in cell shape, the inhibition of adhesion and migration on FN [78]. These alterations are connected with cholesterol regulation of actin cytoskeleton remodeling without any significant alteration of integrin behavior [78]. In Chinese hamster ovary cells (CHO-cells) the alteration of cholesterol at Trans-Golgi/endosome boundaries leads to Syntaxin-6 increase into recycling endosomes influencing the recycling of αvβ3 and α5β1 integrins [79]. Moreover, the same cells overexpressing Annexin 6 are characterized by the inhibition of late endosome cholesterol export and Syntaxin-6 mis-localization that summarize with a reduced cell surface integrin amount [80].

In conclusion, we show how the oncogenic transcription factor TFEB has a relevant impact on the regulation of vascular EC adhesion to the ECM through multiple mechanisms. In particular, beyond its canonical function in lysosome genesis and receptor degradation, TFEB can impact integrin-mediated EC adhesion through the regulation of integrin internalization and trafficking, and these effects are exerted through the metabolic control of endogenous cholesterol synthesis and CAV-1 pro-endocytic function.

## Materials and methods

### Reagents

The following reagents were used in this study: High-Capacity cDNA Reverse Transcription kit, TaqMan PCR Universal Master Mix, TaqMan assays, EZ-Link™ Sulfo-NHS-SS-Biotin, MaxiSorp 96-well plates, phalloidin, DAPI and TO-PRO-3-Stain obtained from Thermo Fisher Scientific; streptavidin-agarose beads obtained from Upstate Biotechnology; [^3^H]-acetate (3600 mCi/mmol) purchased from Amersham Bioscience (Little Chalfont, UK); fluorometric Cholesterol/ Cholesteryl Ester Assay Kit—Quantitation was obtained from Abcam (Cambridge, UK); and [^3^H]-cholesterol (7 Ci/mmol) procured from PerkinElmer (Walthman, MA); Empty Columns PD-10 and Gelatin Sepharose 4B (GE Healthcare).

All other reagents were obtained from Merck KGaA.

### Antibodies

The following antibodies were used in this study: anti-integrin β1 (JB1B) (Abcam); anti-β1 integrin, anti-LAMP1, anti-Integrin alpha5 antibody (SNAKA-51) and phalloidin-Atto (Merck KGaA); anti-β-tubulin, anti-paxillin (5H11), SCAP, SREBP2, HMGCR, Alexa Fluor-tagged secondary antibody (Thermo Fisher Scientific); anti-active CD29 (9EG7) (BD Biosciences); anti-TFEB (MyBioSource); anti-CAV-1 and Mouse Mab anti-ED-A Fibronectin (ED-A FN) IST-9 (Santa Cruz Biotechnology); peroxidase-conjugated AffiniPure Rabbit-anti-Goat-IgG, Goat-anti-Mouse IgG and Goat-anti-Rabbit IgG (Jackson ImmunoResearch Laboratories).

### Cells and genetic manipulation

The experiments were performed using human endothelial cells (HUVECs) isolated from umbilical-cord veins maintained as described previously [25]. To minimize cell variability, pools of five different donors were used [81]. The isolation of primary HUVECs was approved by the Office of the General Director and Ethics Committee of the Azienda Sanitaria Ospedaliera Ordine Mauriziano of Torino Hospital (protocol approval no. 586, Oct 22 2012 and no. 26884, Aug 28 2014, no. 1494 July 9 2018 and no. 0005355 May 03 2021), and informed consent was obtained from each patient. The cells were tested for mycoplasma contamination using a Venor GeM Mycoplasma Detection kit.

Loss-of-function experiments were performed with scramble control shRNA or shRNA against TFEB (shRNA #1: TRCN0000013111, shRNA #2: TRCN0000013110 and shRNA #3: TRCN0000437246 NM_007162.2) cloned in the pLKO.1-puro non-Mammalian vector. These cells were named scr-shRNA-ECs and sh-TFEB-ECs #1 or sh-TFEB-ECs #2 or sh-TFEB-ECs #3, respectively. We verified the efficiency, specificity and the putative off-targets effects of the different sh-RNAs against TFEB not finding any difference between them as shown in Fig S1. Since in previous works we had used the sh-RNA #1 [25] it was chosen again for the development of the entire study. HUVECs were transduced with specific lentiviral particles (MOI = 1) prepared according to Follenzi et al. [82]. The medium was replaced after 24 h, and cells stably expressing the lentivirus were selected on puromycin (1 μg/ml) for 24 h. As previously described [25], we verified the upregulation or inhibition of TFEB in ECs by qPCR, immunofluorescence and immunoblotting analyses.

SiRNA-mediated HMGCR silencing in ECs was performed by the use of RNAiMAX lipofectamine in Optimem medium according to the manufacturer’s protocol. Loss-of-function experiments were performed with commercial scramble control siRNA negative control #1 or siRNA against HMGCR (60 nM, 24 h) (SASI_Hs01_00014353; SASI_Hs01_00014354; SASI_Hs01_00014355; SASI_Hs02_00303376). We verified the inhibition of HMGCR in ECs by qPCR and immunofluorescence analysis.

### Adhesion assay

HUVECs (10 × 10^4^ cells/well, six wells per condition) were seeded in 20% FBS medium in 96-well plates coated with fibronectin (3 µg/ml). After 2 h of incubation at 37 °C in the presence of 5% CO_2_, the not-adherent cells were washed away with PBS, and the adherent cells were fixed in 2.5% glutaraldehyde for 30 min and stained with 0.1% crystal violet. Four random fields of each sample were counted at 10 × magnification.

### Integrin recycling assay

Integrin internalization assays were performed as previously described [36]. HUVECs were PM-labeled at 4 °C with 0.5 mg/ml sulfo-NHS-SS-biotin in PBS for 30 min on ice. The labeled cells were washed with cold MEM with 1% FBS and cold PBS, and endocytosis was induced with prewarmed MEM, 1% FBS. At the indicated times, the cells were transferred to ice, and biotin was removed from the plasma membrane by incubation with 20 mM sodium 2-mercaptoethanesulfonate (MesNa) in 50 mM Tris–HCl (pH 8.6), 100 mM NaCl, and 0.015 N NaOH for 1 h at 4 °C. MesNa was quenched by the addition of 20 mM iodoacetamide for 10 min. To measure integrin degradation in ECs we induced internalization as previously described and then cells were lysed after the different times. The cells were lysed in a specific buffer (25 mM Tris–HCl, pH 7.6, 100 mM NaCl, 2 mM MgCl_2_, 1 mM Na_3_VO_4_, 0.5 mM EGTA, 1% Triton X-100, 5% glycerol, and protease and phosphatase inhibitor cocktail) at 4 °C. The lysates were cleared by centrifugation at 12,000 g for 20 min, and the levels of biotinylated integrin were determined by capture-ELISA.

### Capture ELISA

Corning 96-well Clear Polystyrene High Bind Stripwell Microplates were coated overnight with 5 μg/ml specific anti-integrin antibodies in 0.05 M Na_2_CO_3_ (pH 9.6) at 4 °C and then blocked in PBS containing 0.05% Tween-20 with 5% BSA for 1 h at RT.

Integrins were captured by overnight incubation of 50 μl of cell lysate at 4 °C. The unbound material was removed by washing with PBS-Tween. Biotin-integrin complexes were bound with streptavidin-conjugated horseradish peroxidase in PBS-Tween with 1% BSA for 1 h at 4 °C and detected through a chromogenic reaction with ortho-phenylenediamine. The percentage of integrin internalization was calculated as the percentage of biotin-integrin complexes in cell lysates after internalization stimulation with respect to all the biotin-integrin complexes in unstimulated cells.

### Quantification of immunofluorescence analysis

The cells were plated on 0.17-mm glass coverslips (no. 1.5) coated with fibronectin (3 µg/ml), in PBS and allowed to adhere overnight. The cells were washed in PBS, fixed in 4% PFA, permeabilized in 0.1% Triton X-100 for 8 min at 4 °C or with 0,1% saponin in PBS for 5 min at 4 °C, saturated with 1% donkey serum in PBS (30 min) and incubated with specific primary antibodies for 1 h at RT and then with appropriate Alexa Fluor-tagged secondary antibodies.

For evaluation of the plasma membrane integrin amount and fibrillogenesis, living cells on coverslips were incubated with anti-β1 integrin (JB1B), anti-active β1 integrin (9EG7) anti-anti-active α5β1 integrin (SNAKA-51) antibodies labeling the extracellular domain of the protein for 20 min at 37 °C, washed with PBS, fixed, permeabilized, stained with Alexa Fluor antibody and then processed for immunofluorescence detection.

A Leica TCS SP8 gated stimulated emission depletion (g-STED) 3 × laser-scanning microscope was used to acquire super-resolved images (Leica Microsystems). Different fields (5–8) from each sample section were randomly chosen for analysis. When evaluating the same molecule in different samples, the laser power, gain and offset settings were maintained. The images were quantified using ImageJ software.

The integrin amount in lysosomes, early endosomes was analyzed using ImageJ by creating a mask around the LAMP-1 + , EEA1 + or CAV-1 + vesicles and measuring the mean intensity of integrin in the area identified by the mask. The inhibition of endosome acidification was performed by the incubation of ECs with bafilomycin A1 (30 nM, 24 h).

For quantification of the FA and fibrillar adhesion number, area and Feret’s factor, all the images were thresholded using an empirically determined value that was selected to better identify adhesive structures and exclude nonspecific noise. This value was maintained constant for all the images obtained with the different conditions and experiments. Using the ImageJ shape descriptor aspect ratio (AR), we identified the paxillin-positive objects by selecting only those that displayed a major-to-minor axis ratio < 5 per 100 μm^2^ of cell area, whereas for fibrillar adhesions, we identified the anti-active β1 integrin (9EG7)-positive objects based on a major-to-minor axis ratio ≥ 1 per 100 μm^2^ of cell area.

TIRF microscopy was performed using a Leica AM TIRF MC system mounted on a Leica AF 6000LX workstation with a 63X oil-immersion objective and a laser penetration depth of 90 nm. ECs were incubated with anti-active β1 integrin (9EG7) antibodies for 20 min at 37 °C, were fixed, saturated and permeabilized, and then treated with Ab anti-CAV-1 and appropriate Alexa fluor secondary Ab.

### FN fibrillogenesis

Cells were seeded in 6-well dishes at a concentration of 4 × 10^5^ cells per well and left to adhere for 3 or 16 h medium containing FN-depleted serum were incubated with IST-9 antibody, washed with PBS, fixed with 4% PFA and processed for immunofluorescence. The amount of ED-A FN fibrils was quantified. FN-depleted FCS was prepared according to previously described protocol [35] by the use of gelatin-Sepharose 4B.

### Real-time PCR

Extracted RNA was converted to cDNA using a High-Capacity cDNA Reverse Transcription kit. Real-time PCR was performed using a CFX96 system (Bio-Rad) with TaqMan PCR Universal Master Mix and specific TaqMan assays. The experiments were performed in triplicate, and *TBP* was used as a reference gene.

The following TaqMan assays were used: TFEB (Hs00292981_m1), ITGB1 s01127536_m1), ITGB3 (Hs01001469_m1), ITGA5 (Hs01547673_m1), ITGAV (Hs00233808_m1), CAV-1 (Hs00971716_m1), SCAP (Hs00378725_m1), SREBF-2 (Hs01081784_m1), HMGCR (Hs00168352_m1), and TBP (Hs00427620).

### Western blot

Western blotting and quantitative analysis were performed using the specific above-mentioned antibodies, a ChemiDoc Touch Imaging System (Bio-Rad) and Image Lab software 5.2.1 (Bio-Rad) as previously described [25].

### Immunoprecipitation

Cells were washed with cold PBS and lysed in buffer with added protease and phosphatase inhibitors (50 μg/ml pepstatin, 50 μg/ml leupeptin, 10 μg/ml aprotinin, 1 mM phenylmethylsulfonyl fluoride, 100 μM ZnCl_2_, 1 mM Na_3_VO_4_). To immunoprecipitate integrin complexes, a buffer containing 50 mM HEPES (pH 7.4), 5 mM EDTA, 2 mM EGTA, 150 mM NaCl, 10% glycerol, and 1% NP-40 was used. For all of the other conditions of immunoprecipitation, a buffer containing 10 mM Tris–HCl pH 7.5, 150 mM NaCl, 5 mM EDTA, 1% Triton X-100, 10% glycerol was used. Lysates (1 mg) were precleared for 2 h at 4 °C and then incubated with protein G-Sepharose or protein A-Sepharose and anti-active β1 integrin (9EG7) or active α5β1 integrin (SNAKA-51) or anti-β1 integrin antibodies for 2 h at 4 °C. After resuspension in 50 mM Tris–HCl pH 7.4, 2% SDS and heated for 5 min at 95 °C, immunoprecipitates were resolved by SDS-PAGE and immunoblotted as indicated. In the same lysates used for immunoprecipitation, the endogenous expression of total β1 integrin and β-tubulin were blotted with the specific antibody and presented as “input”.

### De novo synthesis of isoprenoids and cholesterol

Cells (0.5 × 10^6^) were grown in medium containing 10% FBS, radiolabeled with 1 μCi [^3^H]-acetate for 24 h and then washed twice with PBS. A 50-μl aliquot was sonicated and used to measure the protein content, and the remaining cell suspension was subjected to 1:2 methanol/hexane lysis for the collection of lipid species. Lipids resuspended in 30 μl of chloroform were separated by thin layer chromatography (TLC) using a 1:1 (v/v) ether/hexane solution as the mobile phase and LK6DWhatman silica gels (Merck, Darmstadt, Germany). Solutions of 10 mg/ml cholesterol, ubiquinone, FPP or GPP were used as standards. The silica gel plates were exposed for 1 h to an iodine-saturated atmosphere, the migrated spots were cut out, and their radioactivity was measured by liquid scintillation using a Tri-Carb Liquid Scintillation Analyzer (PerkinElmer, Waltham, MA, USA) [83]. The results were determined using previously prepared calibration curves and are expressed as pmoles/mg cellular proteins.

### Free cholesterol quantification

The fluorometric Cholesterol/Cholesteryl Ester Assay Kit—Quantitation was used to measure the level of free cholesterol in cell lysates in accordance to the manufacturer’s instructions. The results are expressed as mg cholesterol or cholesteryl esters/mg cellular proteins.

### Cholesterol uptake and efflux

Cells (0.5 × 10^6^) were grown in medium containing 10% FBS and radiolabeled with 1 μCi [^3^H]-cholesterol (7 Ci/mmol). In the uptake assay, the medium was removed after 0.5 h, and the cells were washed five times with PBS and detached. The radioactivity of the cell suspension, which is a measure of cholesterol uptake, was measured by liquid scintillation. In the efflux assay, the medium was removed after 1 h, and the cells were washed five times with PBS and allowed to grow for an additional 24 h. After this incubation time, the extracellular medium was collected, and the radioactivity, which serves as an index of cholesterol efflux over 24 h, was determined by liquid scintillation [83]. The count per minute (cpm) of scr-shRNA-ECs-treated cells was considered 100%, and the results are expressed as the percentages of the cpm of the experimental cells versus that of the scr-shRNA-treated ECs.

### Statistical analysis

The sample sizes were not selected according to a specific power analysis but were consistent with those used in similar experiments performed in other laboratories investigating vascular development and quoted in the specific references. No statistical methods were used to predetermine the sample size. We did not randomize the samples because our experimental design did not require this type of strategy. The investigators were not blinded to the allocation of the samples during the experiments and analyses. The data are presented as the mean ± SEM.

The statistical analyses were performed using Excel (Microsoft) and Prism (GraphPad) software. Appropriate statistical tests were performed as indicated in the Results section, and *p* < 0.05 was considered to indicate statistical significance in all the experiments.

## Supplementary Information

Below is the link to the electronic supplementary material.

Supplementary file1 (PDF 9872 kb) Silencing of TFEB and modulation integrins, FAs/ FBs development, cell adhesion by the use of different specific sh-RNAs in ECs. **A** qPCR of *TFEB* expression in scr-shRNA-ECs and sh-TFEB-ECs #1, sh-TFEB-ECs #2 and sh-TFEB-ECs #3 respectively infected with 3 different specific lentiviral TFEB sh-RNAs. The data are expressed as the relative fold changes in sh-TFEB-ECs compared with the expression in scr-shRNA-ECs after normalization to the housekeeping gene *TBP* (*n* = 3 independent experiments; values as mean ± SEM; ***p< 0.0001 for sh-TFEB- *versus* scr-shRNA-ECs, as determined by Student’s *t-*test). **B** Representative western blots of the cellular amounts of TFEB and total β1 integrin expression in scr-shRNA-ECs and in sh-TFEB-ECs #1, sh-TFEB-ECs #2 and sh-TFEB-ECs #3 respectively infected with 3 different specific TFEB lentiviral sh-RNAs. ** C** Representative images of adherent scr-shRNA-ECs and sh-TFEB-ECs #1, sh-TFEB-ECs #2 and sh-TFEB-ECs #3 respectively infected with 3 different specific lentiviral TFEB sh-RNAs. Cells were seeded on FN (10 × magnification, scale bar: 200 µm). **D** Confocal microscopy analysis of scr-shRNA-ECs and sh-TFEB-ECs #1, sh-TFEB-ECs #2 and sh-TF EB-ECs #3 respectively infected with 3 different specific lentiviral TFEB sh-RNAs stained with anti-paxillin Ab (scale bar: 25 µm). **E** Confocal microscopy analysis of active-β1 integrin and tensin co-localization in living scr-shRNA- and sh-TF EB-ECs following incubation with anti-active β1 integrin (9EG7) and tensin (scale bar: 25 µm). (Fibrillar adhesions number: scr-shRNA-ECs 168.1±29.4 and sh-TFEB-ECs 383.5±63, *p* = 0.001; fibrillar adhesion average size: scr-shRNA-ECs 0.37±0.1 µm and sh-TFEB-ECs 0.82±0.04, *p* = 0.0001; β1 integrin (9EG7) and tensin co-localization area: scr-shRNA-ECs 109.1±14.3 µm^2^ and sh-TFEB-ECs 301.4±48.3 µm^2^, *p* = 0.003; values as mean ± SEM; p as determined by Student’s *t-*test; *n* = 20 cells per condition pooled from 3 independent experiments)

Supplementary file2 (PDF 5200 kb) TFEB modulation of active-α5β1-FN pathway in ECs. **A** qPCR of *ITG1B* expression in scr-shRNA-ECs and sh-TFEB-ECs #1, sh-TFEB-ECs #2 and sh-TFEB-ECs #3 respectively infected with 3 different specific lentiviral TFEB sh-RNAs. The data are expressed as the relative fold changes in sh-TFEB-ECs compared with the expression in scr-shRNA-ECs after normalization to the housekeeping gene *TBP* (*n* = 3 independent experiments; values as mean ± SEM; *p* = ns for sh-TFEB- *versus* scr-shRNA-ECs, *p* = ns as determined by Student’s *t-*test). **B** Representative western blot of total β1 and active-α5β1 integrins immunoprecipitated from scr-shRNA- or sh-TFEB-ECs lysates. After immunoprecipitation (IP) with anti-active-α5β1 integrin (SNAKA51) and anti-total β1 integrin antibodies, proteins were blotted (IB) with anti-total β1 integrin antibody.In the same lysates used for immunoprecipitation, the endogenous expression of β-tubulin was blotted with the specific antibody. **C** Representative western blots of cellular amount of ED-A FN expression in scr-shRNA- and sh-TFEB-ECs. Bar graph shows the densitometric analysis expressed as the ratio between ED-A FN and β-tubulin (*n* = 3 independent experiments, values as mean ± SEM; *p* = ns sh-TFEB- *versus* scr-shRNA-ECs by Student’s *t-*test). **D** qPCR of *FN1* expression in scr-shRNA-ECs and sh-TFEB-ECs. The data are expressed as the relative fold changes in sh-TFEB-ECs compared with the expression in scr-shRNA-ECs after normalization to the housekeeping gene *TBP* (*n* = 3 independent experiments; values as mean ± SEM; *p* = ns for sh-TFEB- *versus* scr-shRNA-ECs, *p* = ns as determined by Student’s *t-*test). **E** Confocal microscopy analysis of endogenous ED-A FN expression in scr-shRNA- and sh-TFEB-ECs stained with IST-9 antibody and TO-PRO-3after 3 or 16h of incubation with medium FCS FN depleted (scale bar: 25 µm). The bar graph shows the quantification of the mean intensity of ED-A FN (*n* = 3 independent experiments; values as mean ± SEM; ***p < 0.0001 for sh-TFEB-ECs *versus* scr-shRNA-ECs, as determined by Student’s *t-*test). **F** Representative western blots of cellular amount expression of total and Tyr-phosphorylated FAK and Src proteins in scr-shRNA- and sh-TFEB-ECs. Bar graph shows the densitometric analysis expressed as the ratio between p-Tyr/ total protein and β-tubulin (*n* = 3 independent experiments, values as mean ± SEM; ***p < 0.0001 for sh-TFEB- *versus* scr-shRNA-ECs, as determined by Student’s *t-*test)

Supplementary file3 (PDF 1502 kb) TFEB silencing inhibits active integrin degradation. **A** Confocal microscopy analysis of active β1 and active-α5β1 integrin localization in EEA1^+^ and LAMP-1^+^ vesicles in scr-shRNA-ECs and sh-TFEB-ECs stained with anti-active-β1 integrin (9EG7), anti-active-α5β1 (SNAKA51), anti-EEA1 and anti-LAMP-1 antibodies (scale bar: 50 µm). Bar graph shows the quantification ofthe amount of active β1 and active-α5β1 integrin accumulated in EEA1^+^ and LAMP-1^+^ areas (*n* = 20 cells per condition pooled from three independent experiments, values are mean ± SEM; *p < 0.01, **p < 0.001 scr-shRNA- *versus* sh-TFEB-ECs as determined by Student’s *t-*test). **B** Analysis of the relative degradation of cell surface active β1 integrin in scr-shRNA-ECs and sh-TFEB-ECs at different times from the induction of internalization (8h, 24 h) (*n* = 3 independent experiments, values as mean ± SEM, ***p < 0.0001, **p < 0.001 sh-TFEB ECs *versus* scr-shRNA-ECs as determined by Student’s *t-*test)

Supplementary file4 (PDF 618 kb) TFEB regulation of integrin amount via cholesterol synthesis and lysosomal pathway. **A** -Representative confocal microscopy image of scr-shRNA-ECs and scr-shRNA-ECs + siRNA HMGCR stained with anti-HMGCR antibody (scale bar: 25 µm). -qPCR of *HMGCR* expression in scr-shRNA-ECs and scr-shRNA-ECs + siRNA HMGCR#1 or #2 or #3. The data are expressed as the relative fold changes in scr-shRNA-ECs + siHMGCR compared with the expression in scr-shRNA-ECs after normalization to the housekeeping gene *TBP* (*n* = 3 independent experiments; values as mean ± SEM; ***p < 0.0001 and **p < 0.001 for scr-shRNA-ECs + siHMGCR *versus* scr-shRNA-ECs, as determined by Student’s *t-*test).-Quantification of de novo cholesterol synthesis, total/free cholesterol and cholesteryl estersin scr-shRNA- and in scr-shRNA-ECs + siHMGCR grown in medium containing [3H] acetate (n=3 independent experiments; values as mean ± SEM; ***p < 0.0001, **p < 0.001 and *p < 0.01 for scr-shRNA-ECs + siHMGCR versus scr-shRNA-ECs, as determined by Student’s t-test).**B** Time-course analysis of the relative amounts of internalized total and active β1 and integrin in scr-shRNA- and sh-TFEB-ECs treated or not with βMCD. Integrin internalization was evaluated by integrin internalization assay and capture ELISA assay (*n* = 3 independent experiments, values as mean ± SEM; ***p < 0.0001, **p < 0.001, *p < 0.01 all samples *versus* scr-shRNA-ECs as determined by Student’s *t-*test). **C** Representative western blot of active and total β1 integrins immunoprecipitated from scr-shRNA- or sh-TFEB-ECs lysates. After immunoprecipitation (IP) with anti-active β1 integrin (9EG7) and anti-total β1 integrin antibodies, proteins were blotted (IB) with anti-total β1 integrin antibody. Bar graph shows the densitometric analysis of total and active β1 integrins expression amount in scr-shRNA-ECs treated with bafilomycin A1, βMCD or cholesterol (*n*=3 independent experiments, values as mean±SEM; **p < 0.001 and *p < 0.01 for all sample *versus* scr-shRNA-ECs, and ^#^*p* < 0.01 for sh-TFEB+cholesterol vs sh-TFEB as determined by Student’s *t-*test). **D** The bar graphs show the quantification of total cholesterol, free cholesterol and cholesteryl esters in scr-shRNA- and sh-TFEB-ECs supplemented or not supplemented with cholesterol (24h) (*n* = 3 independent experiments; values as means±SEMs; ***p < 0.0001,**p < 0.001 and *p < 0.01 for all samples versus scr-shRNA-ECs, as determined by Student’s t-test; ###*p* < 0.0001, ##*p* < 0.001 and #*p* < 0.01 for sh-TFEB-ECs supplemented with cholesterol versus sh-TFEB-ECs,

Supplementary file5 (PDF 2276 kb) Exogenous cholesterol abrogates TFEB silencing effects on integrin and FBs development. (A) Confocal microscopy analysis of plasma membrane active-β1 integrin expression and localization in living scr-shRNA- and sh-TFEB-ECs supplemented or not with cholesterol following in vivo incubation with anti-active β1 integrin (9EG7) antibody and TO-PRO-3 (scale bar: 25 µm). The bar graphs show the quantification of the mean intensity of active-β1 integrin (n=10 cells per condition pooled from three independent experiments; values as mean ± SEM; ***p<0.0001 for sh-TFEB-ECs *versus* scr-shRNA-ECs, as determined by Student’s *t-*test; ^###^p<0.001 for sh-TFEB-ECs supplemented with cholesterol *versus* sh-TFEB-ECs, as determined by Student’s *t-*test). (B) Confocal microscopy analysis of active-β1 integrin and tensin co-localization in living scr-shRNA- and sh-TFEB-ECs treated with cholesterol following incubation with anti-active β1 integrin (9EG7) and tensin Abs (scale bar: 25 µm)
