# Supplementary figures and images for: TFEB controls integrin-mediated endothelial cell adhesion by the regulation of cholesterol metabolism
Source: Angiogenesis. 2022 May 11;25(4):471–92. doi: 10.1007/s10456-022-09840-x (PMC9519734; doi:10.1007/s10456-022-09840-x)

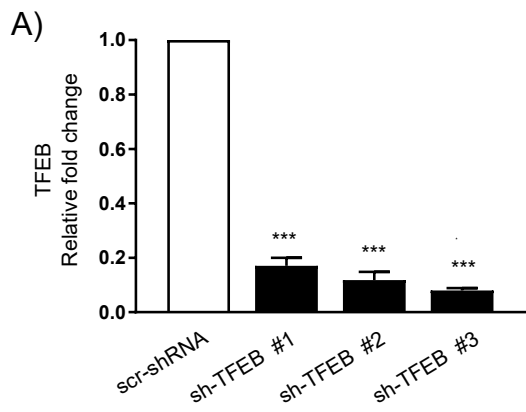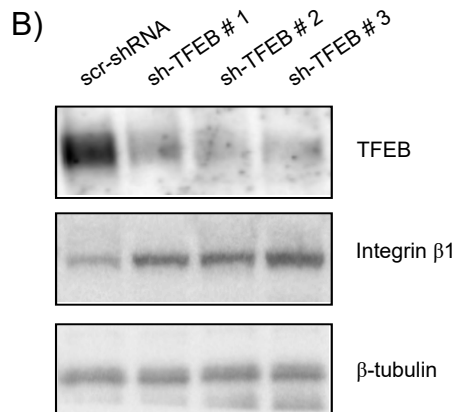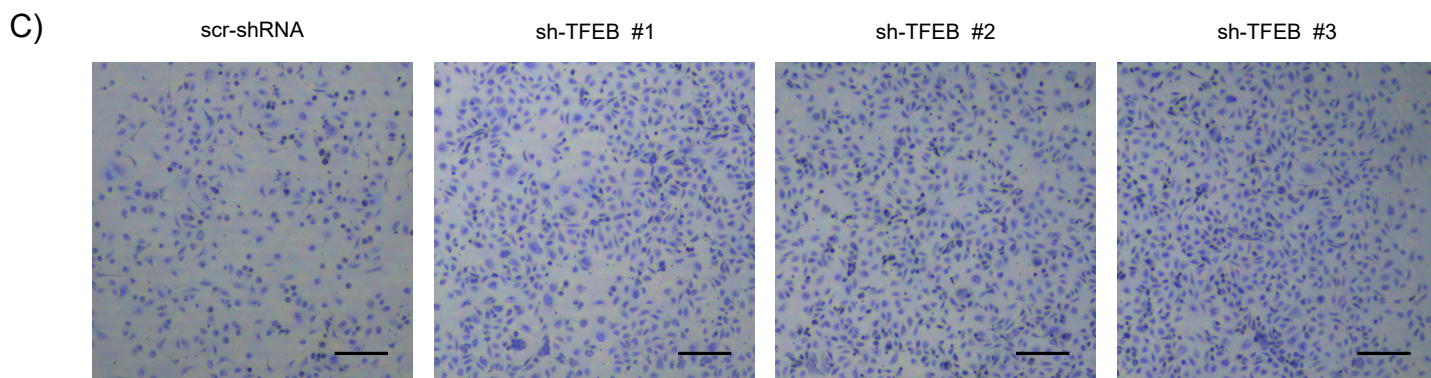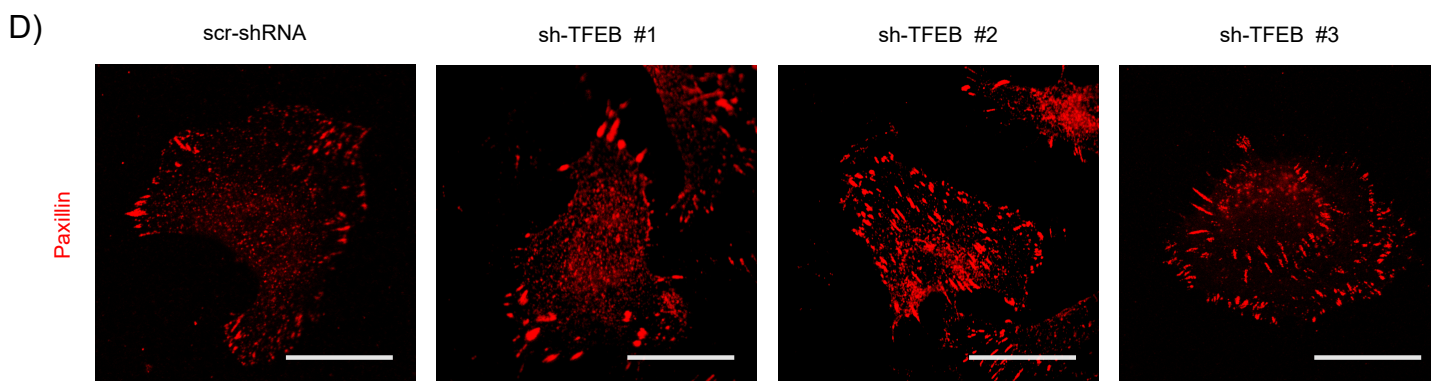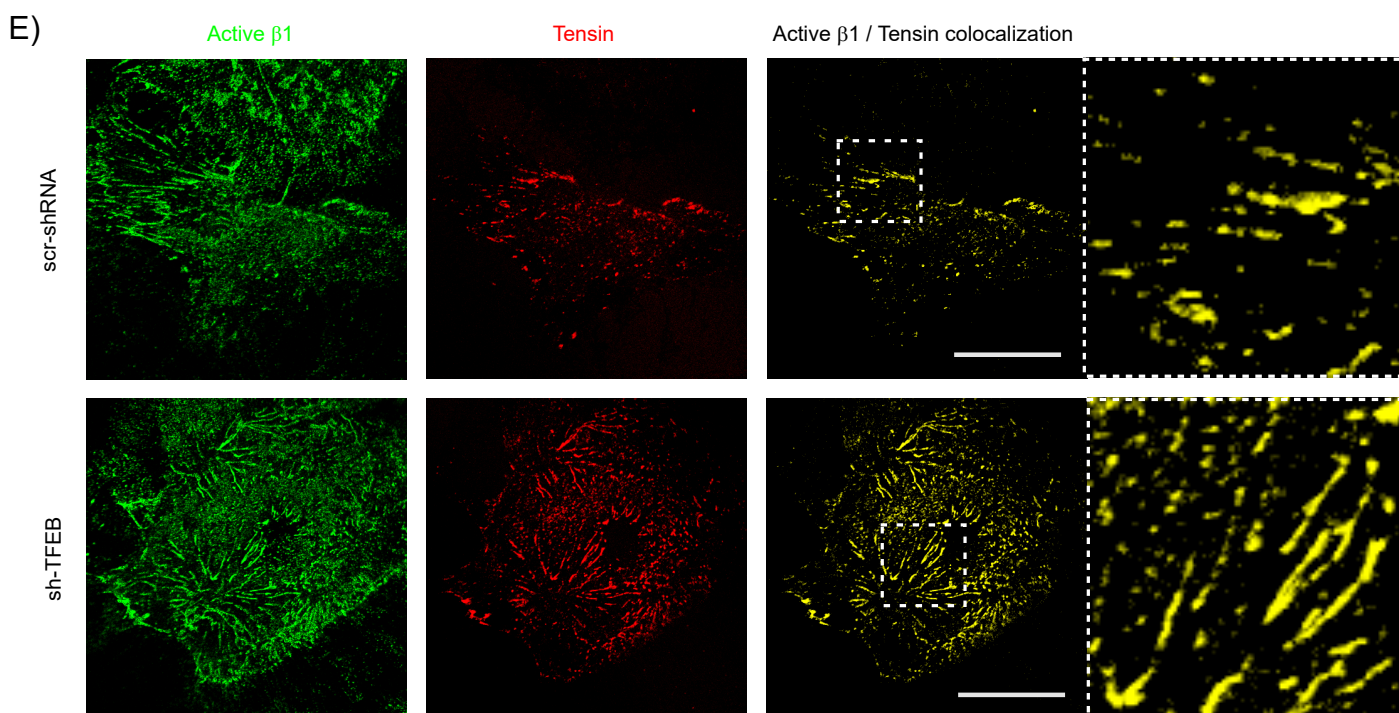

Supplement: Supplementary file 1 — Supplementary file1 (PDF 9872 kb) Silencing of TFEB and modulation integrins, FAs/ FBs development, cell adhesion by the use of different specific sh-RNAs in ECs. A qPCR of TFEB expression in scr-shRNA-ECs and sh-TFEB-ECs #1, sh-TFEB-ECs #2 and sh-TFEB-ECs #3 respectively infected with 3 different specific lentiviral TFEB sh-RNAs. The data are expressed as the relative fold changes in sh-TFEB-ECs compared with the expression in scr-shRNA-ECs after normalization to the housekeeping gene TBP (n = 3 independent experiments; values as mean ± SEM; ***p< 0.0001 for sh-TFEB- versus scr-shRNA-ECs, as determined by Student’s t-test). B Representative western blots of the cellular amounts of TFEB and total β1 integrin expression in scr-shRNA-ECs and in sh-TFEB-ECs #1, sh-TFEB-ECs #2 and sh-TFEB-ECs #3 respectively infected with 3 different specific TFEB lentiviral sh-RNAs. C Representative images of adherent scr-shRNA-ECs and sh-TFEB-ECs #1, sh-TFEB-ECs #2 and sh-TFEB-ECs #3 respectively infected with 3 different specific lentiviral TFEB sh-RNAs. Cells were seeded on FN (10 × magnification, scale bar: 200 µm). D Confocal microscopy analysis of scr-shRNA-ECs and sh-TFEB-ECs #1, sh-TFEB-ECs #2 and sh-TF EB-ECs #3 respectively infected with 3 different specific lentiviral TFEB sh-RNAs stained with anti-paxillin Ab (scale bar: 25 µm). E Confocal microscopy analysis of active-β1 integrin and tensin co-localization in living scr-shRNA- and sh-TF EB-ECs following incubation with anti-active β1 integrin (9EG7) and tensin (scale bar: 25 µm). (Fibrillar adhesions number: scr-shRNA-ECs 168.1±29.4 and sh-TFEB-ECs 383.5±63, p = 0.001; fibrillar adhesion average size: scr-shRNA-ECs 0.37±0.1 µm and sh-TFEB-ECs 0.82±0.04, p = 0.0001; β1 integrin (9EG7) and tensin co-localization area: scr-shRNA-ECs 109.1±14.3 µm2 and sh-TFEB-ECs 301.4±48.3 µm2, p = 0.003; values as mean ± SEM; p as determined by Student’s t-test; n = 20 cells per condition pooled from 3 independent experiments) [file 10456_2022_9840_MOESM1_ESM.pdf]

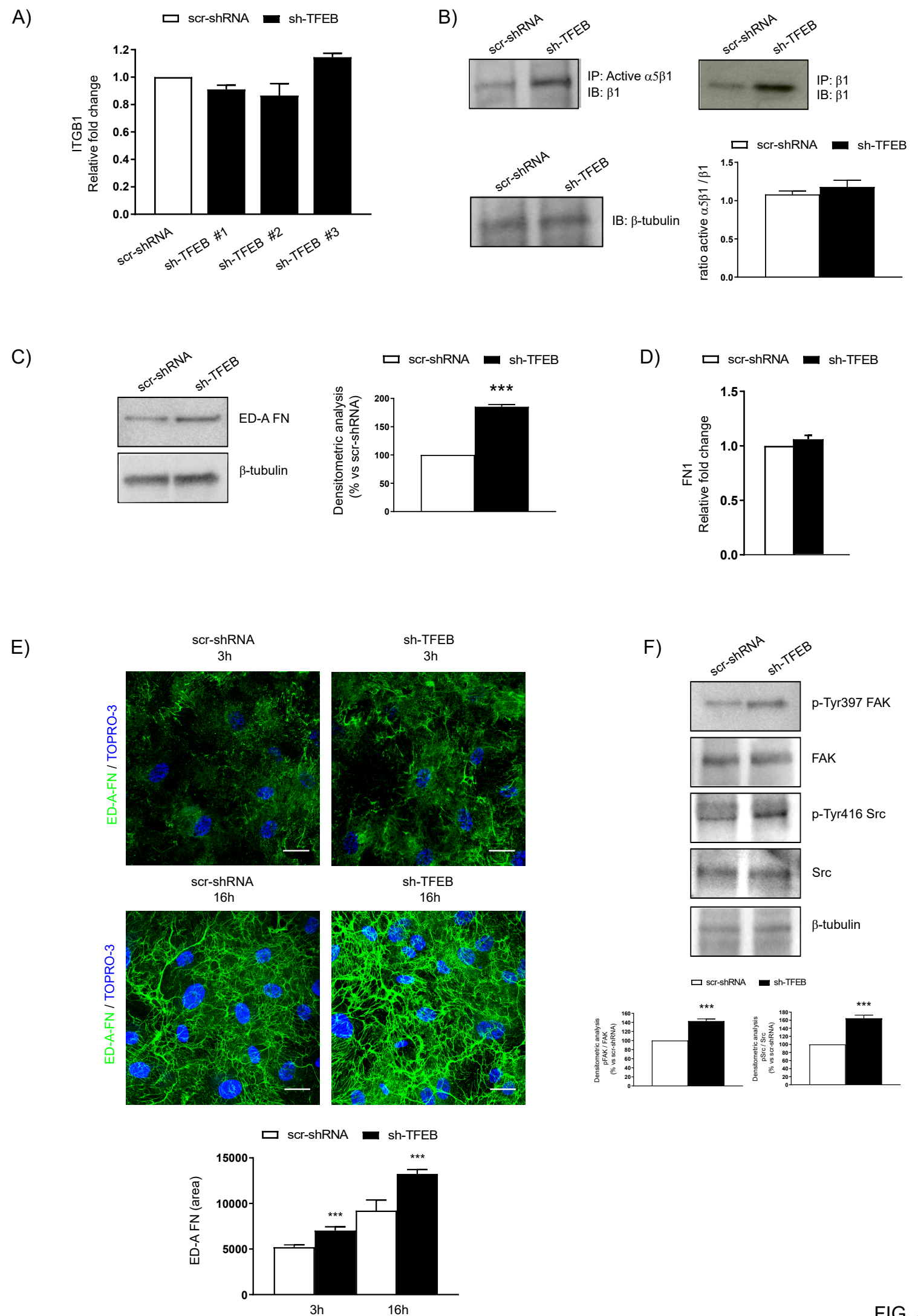

FIG. S2

Supplement: Supplementary file 2 — Supplementary file2 (PDF 5200 kb) TFEB modulation of active-α5β1-FN pathway in ECs. A qPCR of ITG1B expression in scr-shRNA-ECs and sh-TFEB-ECs #1, sh-TFEB-ECs #2 and sh-TFEB-ECs #3 respectively infected with 3 different specific lentiviral TFEB sh-RNAs. The data are expressed as the relative fold changes in sh-TFEB-ECs compared with the expression in scr-shRNA-ECs after normalization to the housekeeping gene TBP (n = 3 independent experiments; values as mean ± SEM; p = ns for sh-TFEB- versus scr-shRNA-ECs, p = ns as determined by Student’s t-test). B Representative western blot of total β1 and active-α5β1 integrins immunoprecipitated from scr-shRNA- or sh-TFEB-ECs lysates. After immunoprecipitation (IP) with anti-active-α5β1 integrin (SNAKA51) and anti-total β1 integrin antibodies, proteins were blotted (IB) with anti-total β1 integrin antibody.In the same lysates used for immunoprecipitation, the endogenous expression of β-tubulin was blotted with the specific antibody. C Representative western blots of cellular amount of ED-A FN expression in scr-shRNA- and sh-TFEB-ECs. Bar graph shows the densitometric analysis expressed as the ratio between ED-A FN and β-tubulin (n = 3 independent experiments, values as mean ± SEM; p = ns sh-TFEB- versus scr-shRNA-ECs by Student’s t-test). D qPCR of FN1 expression in scr-shRNA-ECs and sh-TFEB-ECs. The data are expressed as the relative fold changes in sh-TFEB-ECs compared with the expression in scr-shRNA-ECs after normalization to the housekeeping gene TBP (n = 3 independent experiments; values as mean ± SEM; p = ns for sh-TFEB- versus scr-shRNA-ECs, p = ns as determined by Student’s t-test). E Confocal microscopy analysis of endogenous ED-A FN expression in scr-shRNA- and sh-TFEB-ECs stained with IST-9 antibody and TO-PRO-3after 3 or 16h of incubation with medium FCS FN depleted (scale bar: 25 µm). The bar graph shows the quantification of the mean intensity of ED-A FN (n = 3 independent experiments; values as mean ± SEM; ***p [file 10456_2022_9840_MOESM2_ESM.pdf]

A)

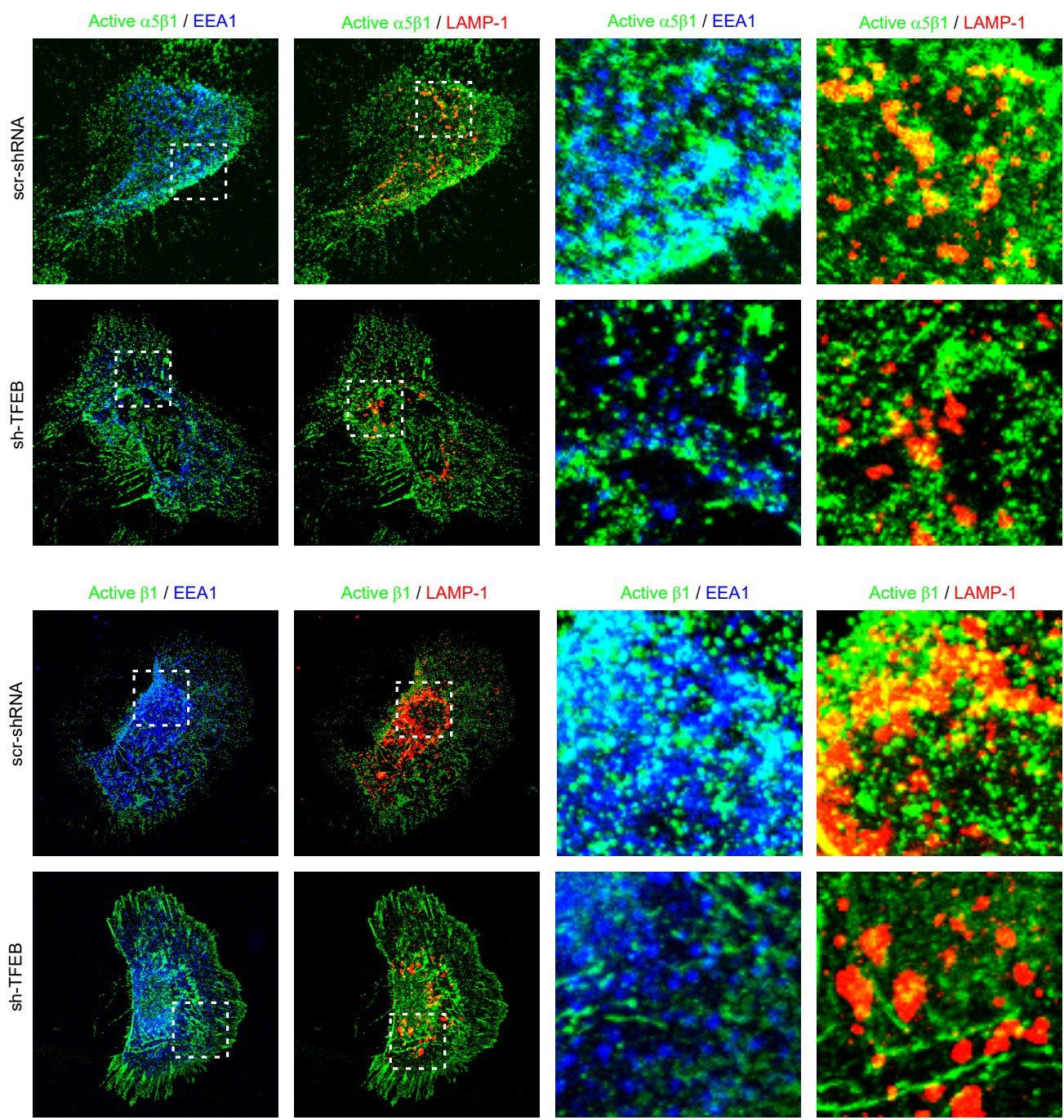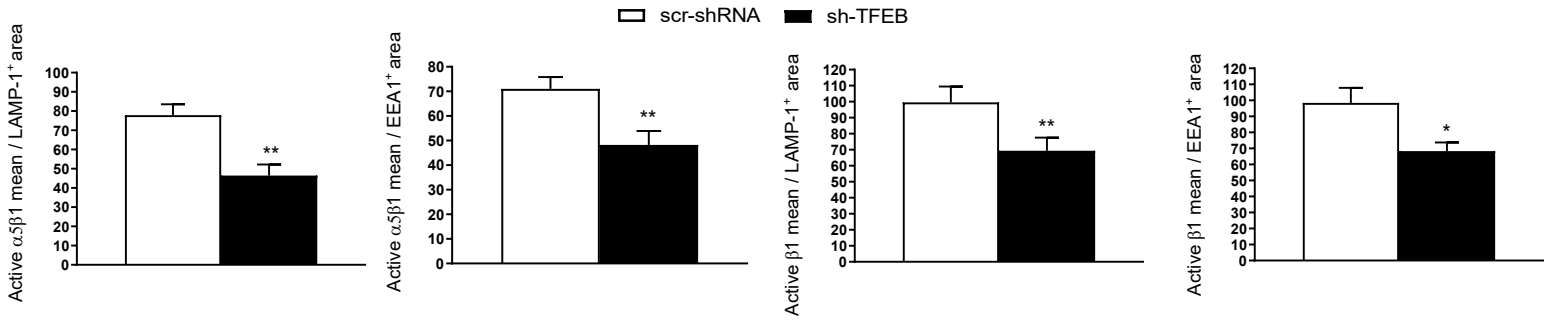

B)

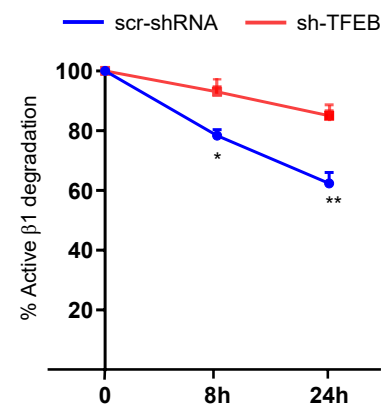

FIG. S3

Supplement: Supplementary file 3 — Supplementary file3 (PDF 1502 kb) TFEB silencing inhibits active integrin degradation. A Confocal microscopy analysis of active β1 and active-α5β1 integrin localization in EEA1+ and LAMP-1+ vesicles in scr-shRNA-ECs and sh-TFEB-ECs stained with anti-active-β1 integrin (9EG7), anti-active-α5β1 (SNAKA51), anti-EEA1 and anti-LAMP-1 antibodies (scale bar: 50 µm). Bar graph shows the quantification ofthe amount of active β1 and active-α5β1 integrin accumulated in EEA1+ and LAMP-1+ areas (n = 20 cells per condition pooled from three independent experiments, values are mean ± SEM; *p < 0.01, **p < 0.001 scr-shRNA- versus sh-TFEB-ECs as determined by Student’s t-test). B Analysis of the relative degradation of cell surface active β1 integrin in scr-shRNA-ECs and sh-TFEB-ECs at different times from the induction of internalization (8h, 24 h) (n = 3 independent experiments, values as mean ± SEM, ***p < 0.0001, **p < 0.001 sh-TFEB ECs versus scr-shRNA-ECs as determined by Student’s t-test) [file 10456_2022_9840_MOESM3_ESM.pdf]

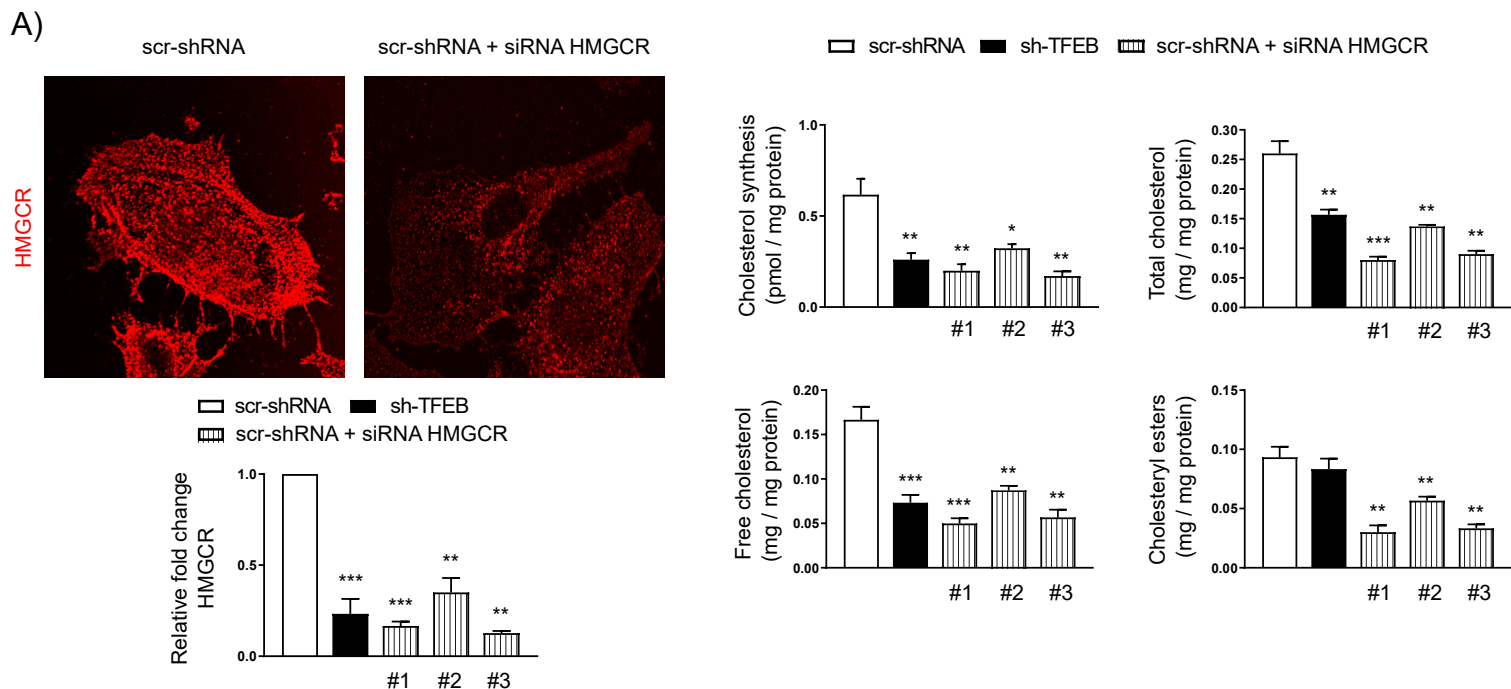

**B)**

scr-shRNA   scr-shRNA+  $\beta$ MCD   sh-TFEB   sh-TFEB +  $\beta$ MCD

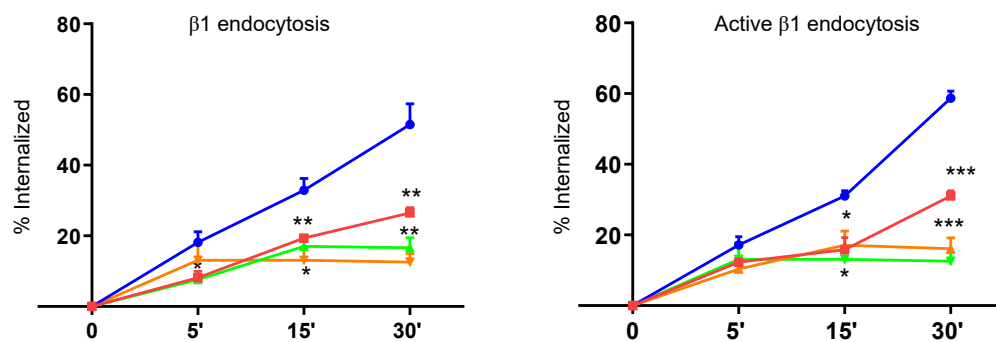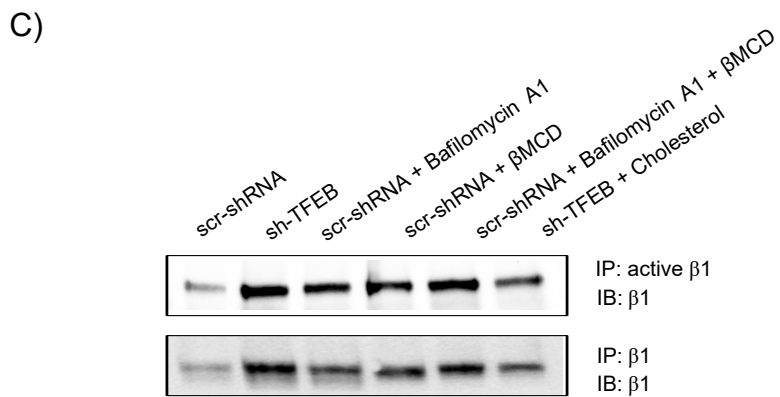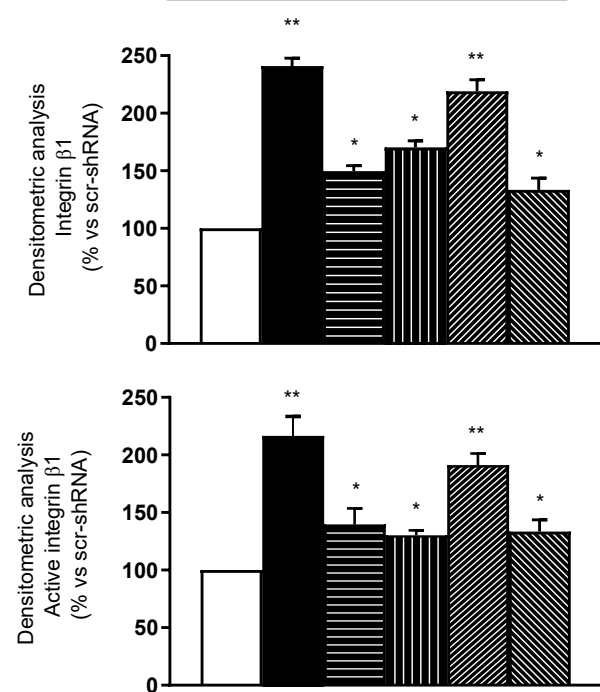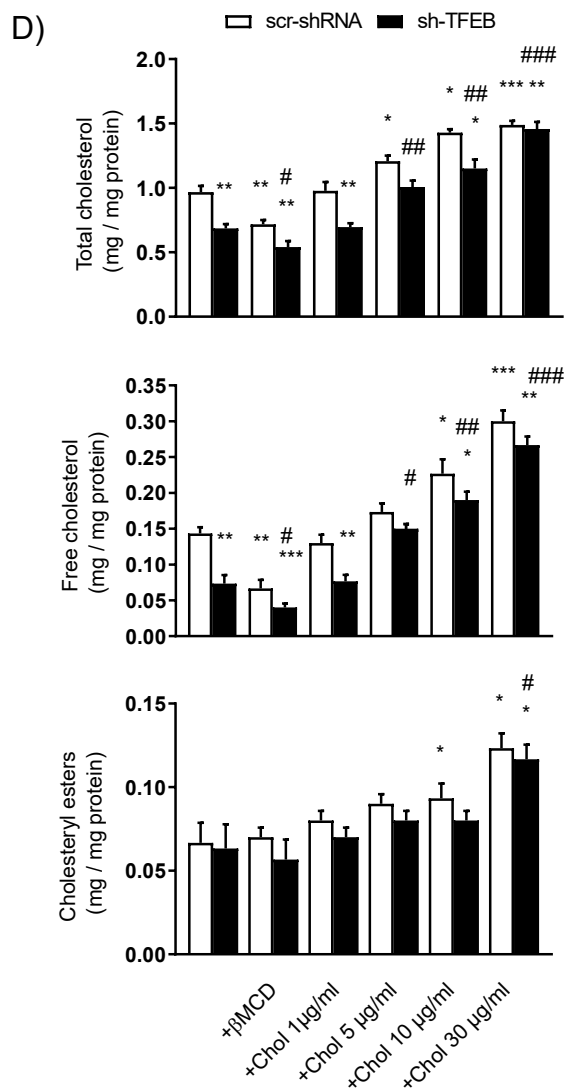

FIG. S4

Supplement: Supplementary file 4 — Supplementary file4 (PDF 618 kb) TFEB regulation of integrin amount via cholesterol synthesis and lysosomal pathway. A -Representative confocal microscopy image of scr-shRNA-ECs and scr-shRNA-ECs + siRNA HMGCR stained with anti-HMGCR antibody (scale bar: 25 µm). -qPCR of HMGCR expression in scr-shRNA-ECs and scr-shRNA-ECs + siRNA HMGCR#1 or #2 or #3. The data are expressed as the relative fold changes in scr-shRNA-ECs + siHMGCR compared with the expression in scr-shRNA-ECs after normalization to the housekeeping gene TBP (n = 3 independent experiments; values as mean ± SEM; ***p < 0.0001 and **p < 0.001 for scr-shRNA-ECs + siHMGCR versus scr-shRNA-ECs, as determined by Student’s t-test).-Quantification of de novo cholesterol synthesis, total/free cholesterol and cholesteryl estersin scr-shRNA- and in scr-shRNA-ECs + siHMGCR grown in medium containing [3H] acetate (n=3 independent experiments; values as mean ± SEM; ***p < 0.0001, **p < 0.001 and *p < 0.01 for scr-shRNA-ECs + siHMGCR versus scr-shRNA-ECs, as determined by Student’s t-test).B Time-course analysis of the relative amounts of internalized total and active β1 and integrin in scr-shRNA- and sh-TFEB-ECs treated or not with βMCD. Integrin internalization was evaluated by integrin internalization assay and capture ELISA assay (n = 3 independent experiments, values as mean ± SEM; ***p < 0.0001, **p < 0.001, *p < 0.01 all samples versus scr-shRNA-ECs as determined by Student’s t-test). C Representative western blot of active and total β1 integrins immunoprecipitated from scr-shRNA- or sh-TFEB-ECs lysates. After immunoprecipitation (IP) with anti-active β1 integrin (9EG7) and anti-total β1 integrin antibodies, proteins were blotted (IB) with anti-total β1 integrin antibody. Bar graph shows the densitometric analysis of total and active β1 integrins expression amount in scr-shRNA-ECs treated with bafilomycin A1, βMCD or cholesterol (n=3 independent experiments, values as mean±SEM; **p < 0.001 and *p < 0.01 for all [file 10456_2022_9840_MOESM4_ESM.pdf]

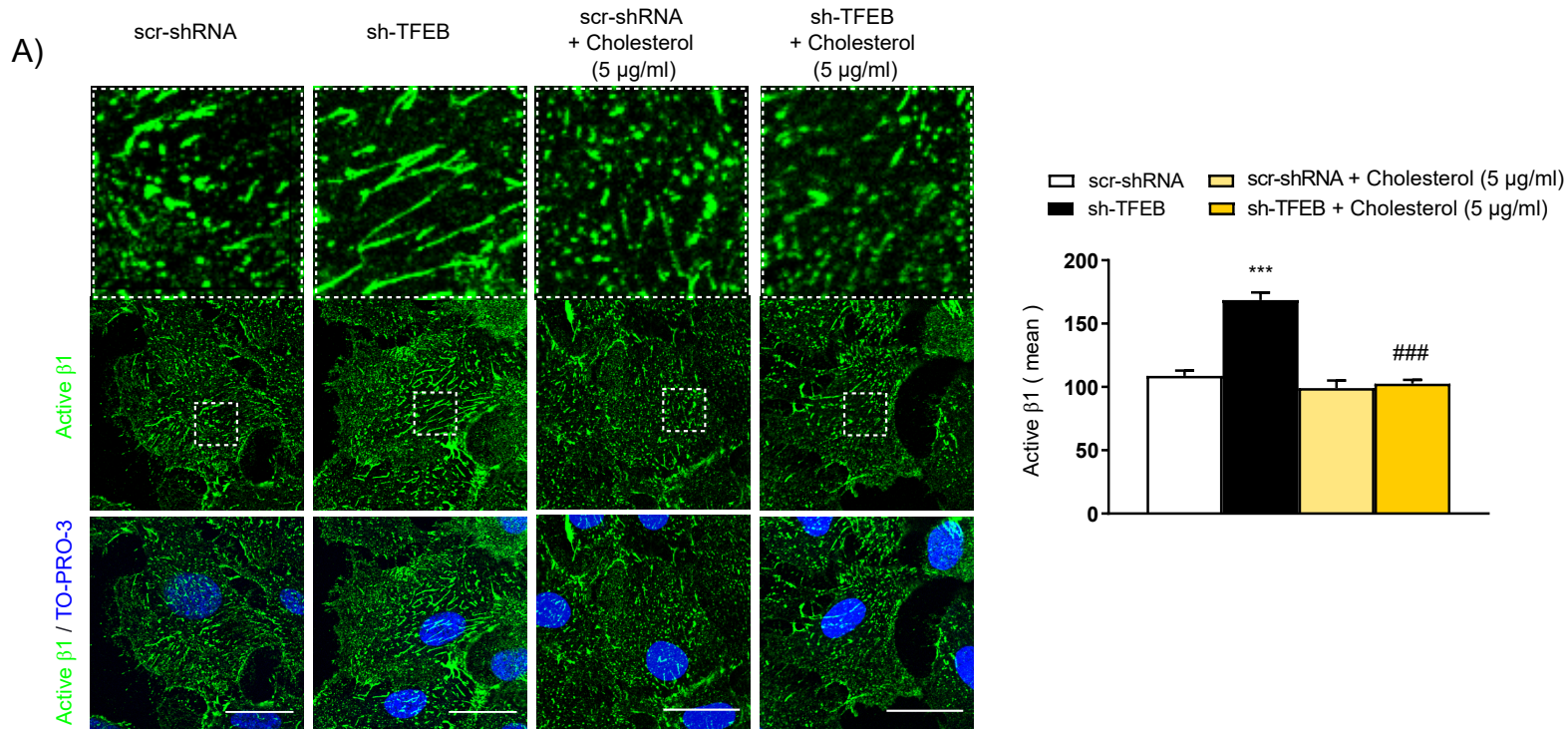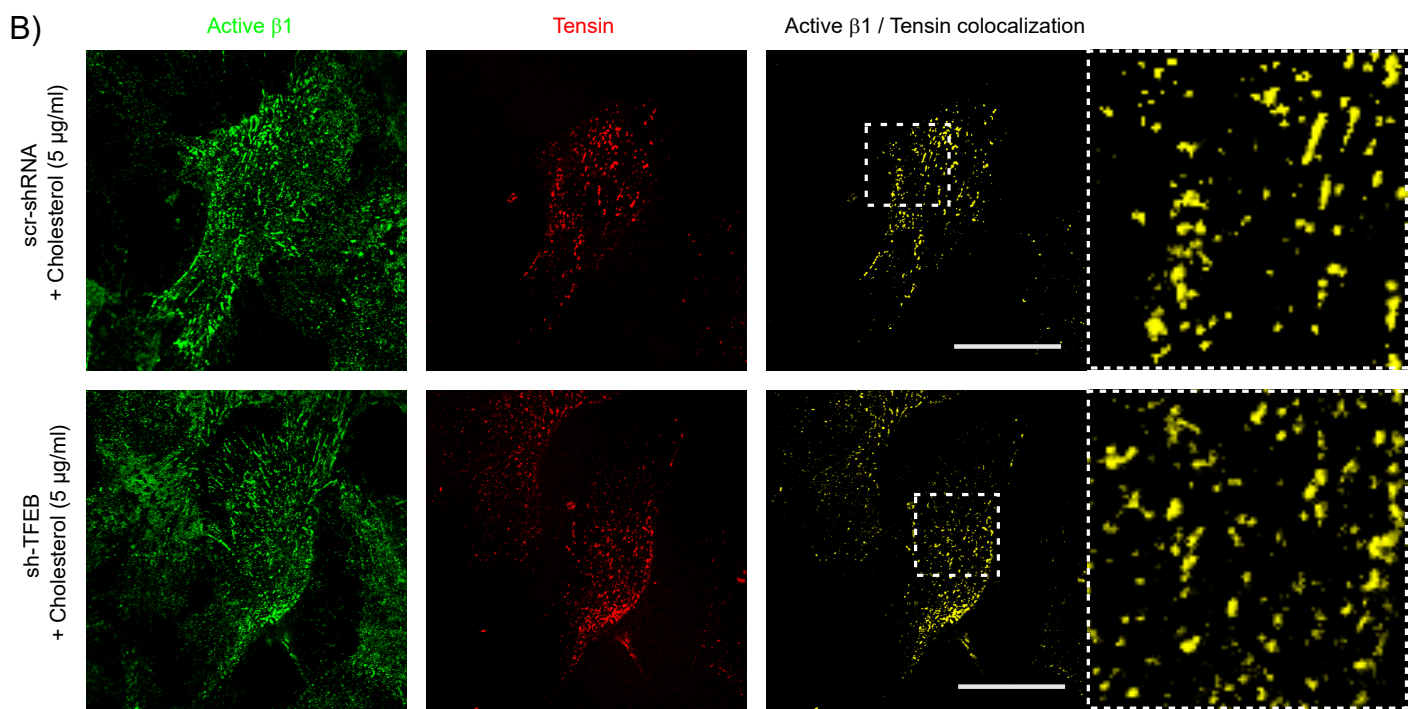

Supplement: Supplementary file 5 — Supplementary file5 (PDF 2276 kb) Exogenous cholesterol abrogates TFEB silencing effects on integrin and FBs development. (A) Confocal microscopy analysis of plasma membrane active-β1 integrin expression and localization in living scr-shRNA- and sh-TFEB-ECs supplemented or not with cholesterol following in vivo incubation with anti-active β1 integrin (9EG7) antibody and TO-PRO-3 (scale bar: 25 µm). The bar graphs show the quantification of the mean intensity of active-β1 integrin (n=10 cells per condition pooled from three independent experiments; values as mean ± SEM; ***p<0.0001 for sh-TFEB-ECs versus scr-shRNA-ECs, as determined by Student’s t-test; ###p<0.001 for sh-TFEB-ECs supplemented with cholesterol versus sh-TFEB-ECs, as determined by Student’s t-test). (B) Confocal microscopy analysis of active-β1 integrin and tensin co-localization in living scr-shRNA- and sh-TFEB-ECs treated with cholesterol following incubation with anti-active β1 integrin (9EG7) and tensin Abs (scale bar: 25 µm) [file 10456_2022_9840_MOESM5_ESM.pdf]
